# Breakpoint Features of Genomic Rearrangements in Neuroblastoma with Unbalanced Translocations and Chromothripsis

**DOI:** 10.1371/journal.pone.0072182

**Published:** 2013-08-26

**Authors:** Valentina Boeva, Stéphanie Jouannet, Romain Daveau, Valérie Combaret, Cécile Pierre-Eugène, Alex Cazes, Caroline Louis-Brennetot, Gudrun Schleiermacher, Sandrine Ferrand, Gaëlle Pierron, Alban Lermine, Thomas Rio Frio, Virginie Raynal, Gilles Vassal, Emmanuel Barillot, Olivier Delattre, Isabelle Janoueix-Lerosey

**Affiliations:** 1 Inserm, U900, Paris, France; 2 Institut Curie, Centre de Recherche, Paris, France; 3 Mines ParisTech, Fontainebleau, France; 4 Inserm, U830, Institut Curie, Paris, France; 5 Centre Léon Bérard, Laboratoire de Recherche Translationnelle, Lyon, France; 6 Institut Curie, Département de Pédiatrie, Paris, France; 7 Institut Curie, Unité de Génétique Somatique, Paris, France; 8 NGS Platform, Institut Curie, Paris, France; 9 Institut Gustave Roussy, Villejuif, France; Duke-National University of Singapore Graduate Medical School, Singapore

## Abstract

Neuroblastoma is a pediatric cancer of the peripheral nervous system in which structural chromosome aberrations are emblematic of aggressive tumors. In this study, we performed an in-depth analysis of somatic rearrangements in two neuroblastoma cell lines and two primary tumors using paired-end sequencing of mate-pair libraries and RNA-seq. The cell lines presented with typical genetic alterations of neuroblastoma and the two tumors belong to the group of neuroblastoma exhibiting a profile of chromothripsis. Inter and intra-chromosomal rearrangements were identified in the four samples, allowing in particular characterization of unbalanced translocations at high resolution. Using complementary experiments, we further characterized 51 rearrangements at the base pair resolution that revealed 59 DNA junctions. In a subset of cases, complex rearrangements were observed with templated insertion of fragments of nearby sequences. Although we did not identify known particular motifs in the local environment of the breakpoints, we documented frequent microhomologies at the junctions in both chromothripsis and non-chromothripsis associated breakpoints. RNA-seq experiments confirmed expression of several predicted chimeric genes and genes with disrupted exon structure including *ALK*, *NBAS*, *FHIT*, *PTPRD* and *ODZ4*. Our study therefore indicates that both non-homologous end joining-mediated repair and replicative processes may account for genomic rearrangements in neuroblastoma. RNA-seq analysis allows the identification of the subset of abnormal transcripts expressed from genomic rearrangements that may be involved in neuroblastoma oncogenesis.

## Introduction

Neuroblastoma (NB), the most frequent extracranial paediatric solid tumor, accounts for 15% of deaths from cancer in childhood and is characterized by a great clinical and genetic heterogeneity. Several types of somatically acquired chromosomal imbalances have been described in this cancer. These include whole chromosome gains or losses, associated with ploidy abnormalities, and structural chromosome alterations (e.g., genomic amplifications or unbalanced translocations) [1]–[3]. Although some regions are recurrently altered (17q, 2p and 1q by gains; 1p, 3p, and 11q by losses), the corresponding breakpoints seem not to be clustered at specific genomic positions but rather scattered along quite large regions [4]–[7]. Importantly, the presence of segmental alterations is strongly associated with a high risk of relapse in NB patients [8]. Moreover, in a subset of cases, it has been documented that tumor progression in NB may be linked to the accumulation of segmental alterations [6].

Whereas a few translocations present in constitutional DNA have been characterized at the gene and base pair level in NB patients [9]–[11], only one unbalanced somatic translocation has been explored at this level in sporadic NB [12]. The full characterization of the der(1)t(1;17) in the CLB-Bar cell line revealed that it was more complex than expected due to the presence of an interstitial 4p telomeric sequence between chromosome 1p and 17q and that three different genes were disrupted by the translocation breakpoints. Interstitial telomere sequences lying at chromosome breakpoints have also been detected in a few other NB cell lines using low resolution cytogenetic techniques [12]. In addition, we previously documented that breakpoints corresponding to unbalanced translocations occur preferentially in early replicating regions in NB cell lines [13], [14]. A larger series of chromosome breakpoints has been analyzed in NB at the sub-kilobase resolution using high-density oligonucleotide microarrays providing the mapping of breakpoints in intervals ranging from 50 bp to 10 kb in size [15]. However, this approach did not offer a full characterization of rearrangements since it did not allow the identification of the various segments involved in a precise rearrangement nor the identification of the breakpoint at the base pair level. More recently, junctions connecting amplified segments involving the *MYCN* gene were characterized in four NB cases [16]. This analysis documented a head-to-tail tandem orientation of the amplicons and frequent microhomology at the junctions.

In the present work, we report an in-depth analysis of two well-characterized NB cell lines and two NB primary tumors using paired-end sequencing on mate-pair libraries. In cell lines, this approach enabled characterization of genomic rearrangements corresponding to simple unbalanced translocations or large intra-chromosomal structural variations (SVs). It also revealed, in a subset of cases, much more complex rearrangements than expected. In one case, the pattern of rearrangements was highly reminiscent of chromothripsis, recently described in several cancer types [17]. This observation prompted us to investigate two primary NB tumors presenting with shattering of one or two specific chromosomes, previously detected by array-CGH. Mate-pair analysis confirmed the geographic localization of the rearrangements and documented their diversity. Using complementary experiments by PCR and Sanger sequencing, we characterized 51 rearrangements in the four samples at the base pair resolution that revealed 59 junctions. Finally, RNA-seq analysis allowed the identification of abnormal transcripts expressed from genomic rearrangements that may be involved in NB oncogenesis.

## Results

### Genomic Rearrangements Revealed by Mate-pair Analysis in NB Cell Lines CLB-Ga and CLB-Re

Paired-end sequencing of mate-pair libraries resulted in 20 and 16 millions of normal pairs (uniquely mapped in outward orientation with insert size around 3 kb, excluding PCR duplicates) for each of the two NB cell lines CLB-Ga and CLB-Re (Table 1, Table S1). This provided an effective coverage of 10X and 8X relative to the six billion-base pair diploid genome in CLB-Ga and CLB-Re cell lines, respectively.

**Table 1:** Sample information.

	Sample type	Diagnosis (D) orRelapse (R)	Diseasestage*	MYCNstatus	Age atdiagnosis		Patient’soutcome
CLB-Ga	cell line	D	4	NA		4 years	Relapse and DOD
CLB-Re	cell line	R	4	A		5 years	Relapse and DOD
NB1141	primary tumor	D	3	A		3.6 years	Relapse and DOD
NB1142	primary tumor	D	2	NA		4 years	Metastatic relapse and CR

A: amplified, NA: not amplified.

* defined according to the INSS classification.

DOD: died of disease.

CR: complete remission.

Reads presenting with abnormal insert size or orientation were further subjected to SVDetect [18]. We identified a number of SVs of the genome and classified them into various categories being inter-chromosomal or intra-chromosomal rearrangements, including deletions, insertions, inversions, inverted duplications, large duplications, etc. In parallel, we generated copy number profiles by applying FREEC [19] on reads that were uniquely mapped to the reference genome (Figure S1).

**Figure 1:**
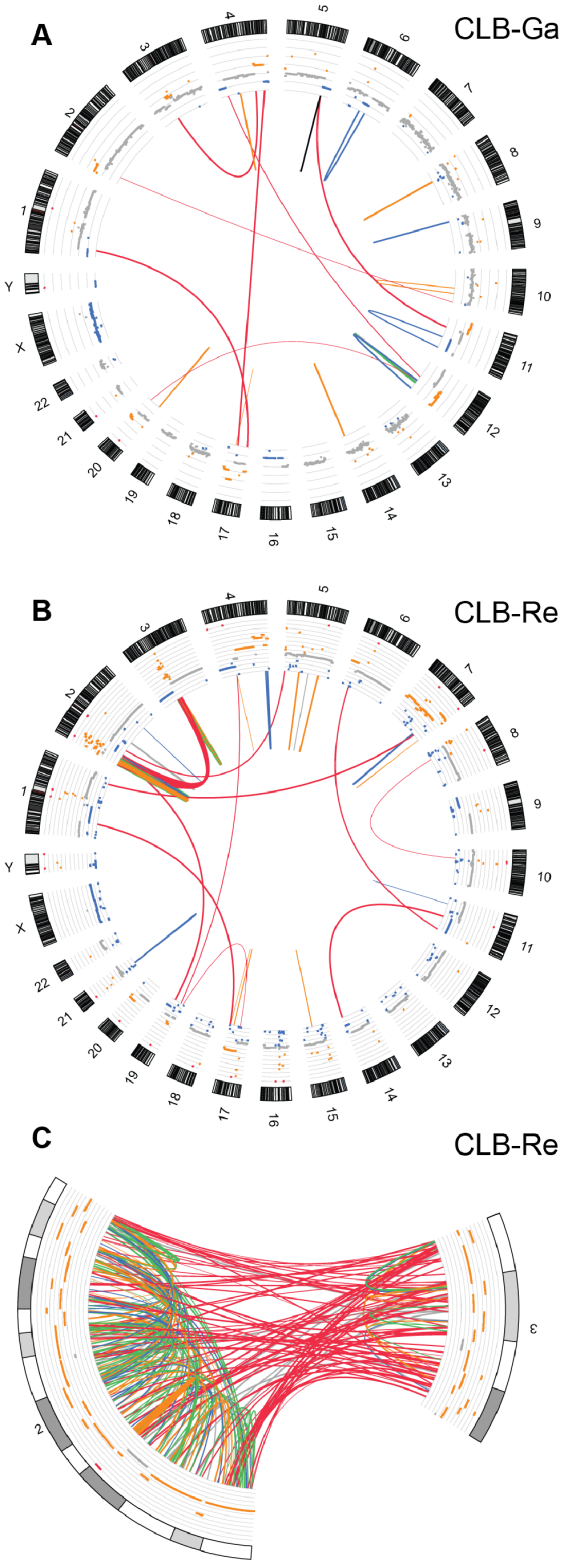
Genome-wide profile of predicted SVs in CLB-Ga and CLB-Re cell lines. All inter-chromosomal rearrangements and intra-chromosomal SVs encompassing regions longer than 50 kb identified in mate-pair sequencing are visualized using Circos [41]. Chromosomes appear as ideograms. The outer ring shows a representation of copy number as determined by sequencing data (grey: normal copy number; blue: deletion, orange: gain). The inner circle shows the two endpoints of each rearrangement identified (black: inverted duplication, blue: deletion, green: inversion, orange: large duplication, red: unbalanced inter-chromosomal rearrangements). Most of the identified unbalanced rearrangements correspond to unbalanced translocations previously identified by 24-color karyotyping. Line’s thickness is related to the numbers of pairs identified in each link. A, CLB-Ga cell line, B, CLB-Re cell line, C, zoom on the cluster of links between chromosomes 2p (0–43.95 Mb) and 3p (58.58–78.99 Mb) in CLB-Re. This dense clustering of SVs was unexpected considering previous characterization of the rearrangements present in this cell line by array-CGH and 24-color karyotyping.

Our mate-pair analysis of the CLB-Ga sample identified 7 inter-chromosomal rearrangements (Figure 1A, Table 2 and Table S2); four of them corresponded to unbalanced translocations previously identified by 24-color karyotyping [14], [20]. The mate-pair analysis documented a rearrangement between chromosome arms 3p and 4q, and another one between chromosome 12q and 20q, not expected from the cytogenetic characterization. In addition, we identified an inter-chromosomal SV between 2p23 and 10q26.3, where the region on chromosome 10 was annotated as a telomeric sequence. We detected 13 large intra-chromosomal rearrangements in the CLB-Ga sample (SV insert size >50 kb), with paired-end mapping (PEM) signatures mostly corresponding to deletions (n = 4) and large duplications (n = 7) (Table 2 and Figure 1). Except for deletions at 6q and 11q, the other predicted rearrangements were not expected from low resolution array-CGH data.

**Table 2:** Inter and large intra-chromosomal rearrangements predicted by SVDetect.

	CLB-Ga	CLB-Re	NB1141	NB1142
Inter-chromosomal links	7	95	7	12
Intra-chromosomal links with an insert size >50 kb	13	175	146	45
Intra-chromosomal links with insert size >50 kb	13	175	146	45
DELETION	4	39	35	11
INS_FRAGMT	0	3	0	1
INV_INS_FRAGMT	0	2	3	3
INV_FRAGMENT	0	8	6	2
INVERSION	1	72	70	16
LARGE_DUPLICATION	7	47	28	10
TRANSLOCATION	1	4	4	2
Inter-chromosomal links	7	95	7	12
Intergenic	3	28	1	2
Promoter	0	1	0	0
Truncated	1	32	3	6
May change function	0	0	0	0
Possible chimera	1	8	2	0
May not change function	0	0	0	0
Does not change function	2	26	1	4
Intra-chromosomal links with an insert size >50 kb	13	175	146	45
Intergenic	3	66	31	11
Promoter	0	3	2	3
Truncated	3	52	50	13
May change function	1	1	0	0
Possible chimera	2	12	15	4
May not change function	0	1	0	0
Does not change function	4	40	48	14

DELETION deletion of a fragment.

INS_FRAGMT insertion of a short fragment (fragment is known).

INV_INS_FRAGMT insertion of a short fragment (fragment is known, fragment is inverted).

INV_FRAGMENT inversion (both ends of the inversion are confirmed by read pairs).

INVERSION inversion (only one end of the inversion is confirmed by read pairs).

LARGE_DUPLICATION large duplication (size of the duplicated fragment is greater than average insert size).

TRANSLOCATION translocation.

For the CLB-Re cell line, mate-pair analysis revealed 95 and 175 inter-chromosomal and large intra-chromosomal rearrangements, respectively (Table 2 and Table S2). Unexpectedly, a high number of inter-chromosomal rearrangements (81/95 = 85%) was detected between a distal portion of chromosome 2p (2 to 44 Mb) and an interstitial portion of chromosome 3p (58 to 78 Mb) (Figure 1B). The distal 2p segment (2pTel to 44 Mb) contained a vast number of large intra-chromosomal SVs (n = 144), compared to chromosome 1 (n = 0) and chromosome 3 (n = 16, all of which fell in the 58–78 Mb interval) (Figure 1C).

Our analysis identified a large number of SVs of short size (SV insert size <50 kb) (Table S2 and Table S3). Since our experiment was designed to focus on large rearrangements, it remains an issue to define the subset of these short SVs that are somatic. Nevertheless, we could determine that these short SVs were mostly deletions (59%) and insertions (30%) and that they fell frequently in intergenic regions (55%) and intronic regions (34%). When comparing these predicted SVs of short size with the Database of Genomic Variants (DGV, hg19, v9) [21], it turned out that 45% of all predicted short SVs were annotated as known SVs in DGV.

### Experimental Validation of Rearrangements and Analysis of Patients’ Samples

**Figure 2:**
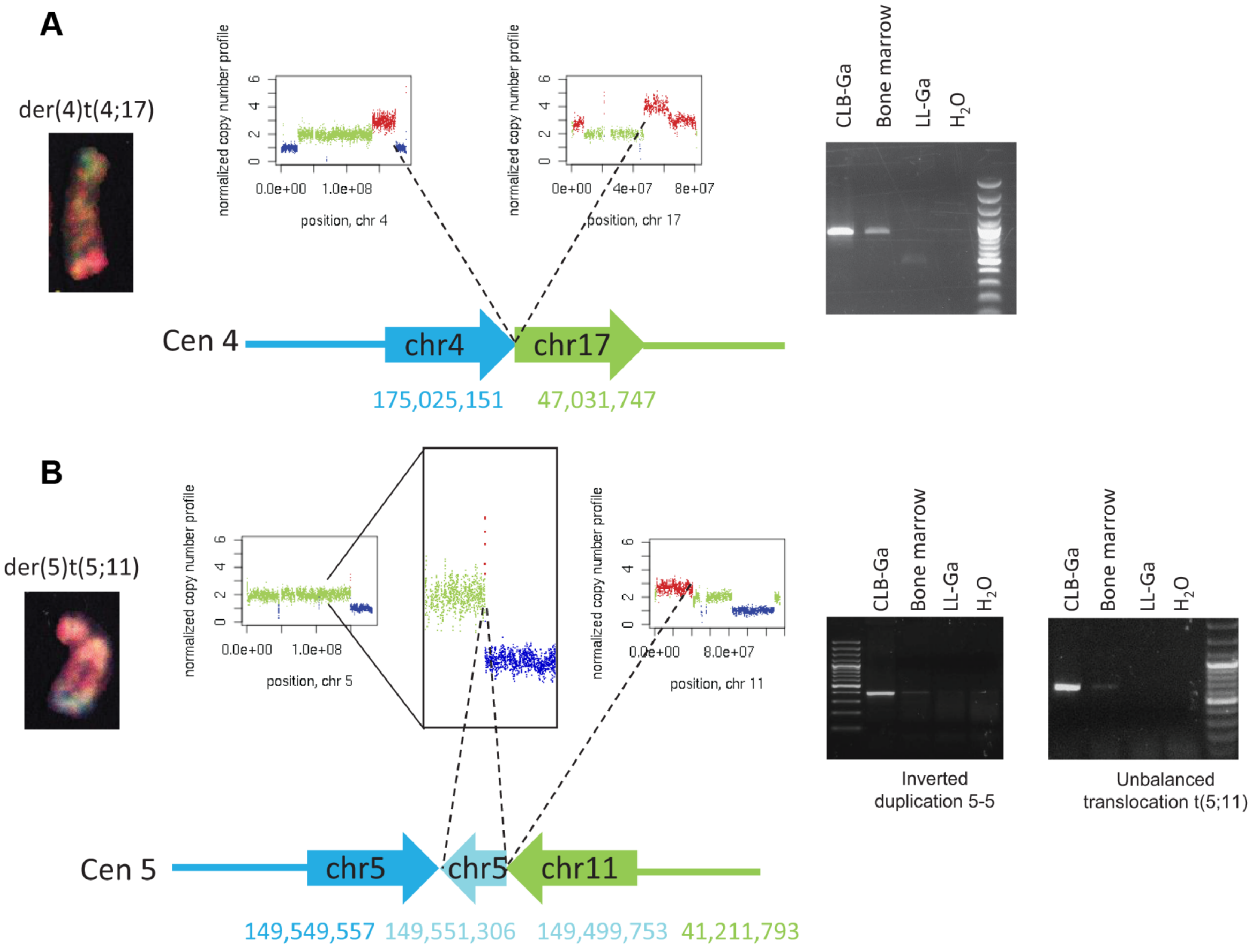
Molecular characterization of unbalanced translocations in the CLB-Ga cell line. **A**, Characterization at the base-pair level of an acquired unbalanced translocation der(4)t(4;17). The translocation was initially demonstrated by 24-color karyotyping (left panel) and loss of 4q as well as gain of 17q was consistently observed by read coverage analysis with the FREEC algorithm (middle panels). NGS data identified abnormally mapped reads corresponding to this inter-chromosomal rearrangement. Further PCR analysis confirmed that this rearrangement was somatic as detected in CLB-Ga cell line as well as in a bone marrow sample contaminated with tumor cells but not in the corresponding lymphoblastoid LL-Ga cell line (right panel). **B**, Characterization at the base-pair level of an acquired unbalanced translocation der(5)t(5;11). The translocation was initially evidenced by 24-color karyotyping (left panel) and loss of 5q as well as gain of 11p was consistently observed by FREEC (middle panels). NGS data identified reads corresponding to an unbalanced translocation between 5q and 11p; however, orientation of the stuck fragments was not compatible with the observed derivative chromosome. NGS data also revealed an intra-chromosomal inverted duplication at 5q close to the rearrangement between 5q and 11p. The FREEC profile indeed revealed a small region of gain preceding the 5q deletion. Further PCR analysis confirmed both rearrangements and indicated that they were somatic as detected in the CLB-Ga cell line as well as in the bone marrow sample contaminated with tumor cells but not in the LL-Ga cell line (right panels). Sequencing of the PCR products indicates that the first breakpoint falls into the first intron of the *CDX1* gene, which is consequently truncated. An inverted duplication is observed between positions 149 499 753 and 149 551 306 comprising the first exon of the *CDX1* gene as well as exons 1 to 17 of the *PDGFRB* gene. The breakpoint at position 41 211 793 on chromosome 11 corresponds to the first intron of the *LRRC4C* gene. Thus, this junction may result in a chimeric transcript including *PDGFRB* and *LRRC4C*.

A subset of rearrangements was selected in each cell line for further experimental validation and analysis of additional patients’ samples. For the CLB-Ga cell line, established at diagnosis (Table 1), we validated by PCR all the seven inter-chromosomal rearrangements identified by the mate-pair analysis. All of them are somatic since they were not detected in the LL-Ga lymphoblastoid cell line. Importantly, these rearrangements could be detected in a bone marrow sample contaminated with tumor cells obtained from the corresponding patient, demonstrating that these events were present in the patient tumor DNA and not acquired in the cell line during the culture process (Figure 2). We also confirmed that the large deletion at 11q was somatic and present at diagnosis in the patient’s tumor cells whereas the deletion at 6q was not detected in such cells suggesting that it has been selected during the culture process (data not shown). In the case of the CLB-Re cell line, we selected 19 inter-chromosomal SVs for validation, including 15 between chromosome arms 2p and 3p, and 4 intra-chromosomal SV, i.e., one deletion predicted at 4q and 3 deletions predicted at 2p. These 23 rearrangements were validated by PCR analysis. Since this cell line has been established at relapse (Table 1), we sought to determine whether such rearrangements were already present in the patient’s tumor at diagnosis. The analysis of 13 rearrangements indicated that they were present in the tumor DNA at diagnosis and absent in the blood DNA (Figure S2 and data not shown).

In order to characterize breakpoints at the base-pair resolution, conventional Sanger sequencing of the PCR fragments was performed followed by a BLAT search [22] on the human reference genome. We characterized 34 rearrangements for both cell lines (Table S4). In the CLB-Ga sample, whereas for translocations der(1)t(1;17), der(4)t(4;17) and der(4)t(4;12), 24-color karyotyping and mate-pair data were fully consistent, the orientation of the fragments involved in the rearrangement between chromosomes 5 and 11 characterized by mate-pair analysis was not compatible with a der(5)t(5;11) chromosome. Detailed examination of the mate-pair data revealed a more complex rearrangement than expected from the cytogenetic study (translocation followed by an inverted duplication, Figures 1A, 2B). Interestingly, the predicted deletion at 12q21.33–12q24.33 that did not match with copy number changes was experimentally validated and shown to be somatic and present in the patient’s bone marrow. This SV is involved in a more complex rearrangement than a simple loss of genomic material at the positions predicted by the mate-pair analysis. It comprises an unbalanced rearrangement between chromosome 12 and 20, together with a gain of a 30 kb region on 12q21.33 and a short pre-telomeric region on 12q24.33 (Figure S3).

### Mate-pair Analysis Confirmed Chromothripsis in Primary Tumors NB1141 and NB1142

The localization of the rearrangements detected in the CLB-Re cell line was compatible with the recently described chromothripsis phenomenon [17]. Yet the copy number changes observed on the implicated segments in that sample did not fulfill the criteria defined for chromothripsis. However, during our previous analysis of a large series of NB tumors by array-CGH we noticed two cases, NB1141 and NB1142, presenting with shattering of a specific chromosome suggesting potential chromothripsis. Therefore, these two cases (Table 1) were subjected to mate-pair analysis. The number of predicted inter- and large intra-chromosomal rearrangements is provided in Table 2.

**Figure 3:**
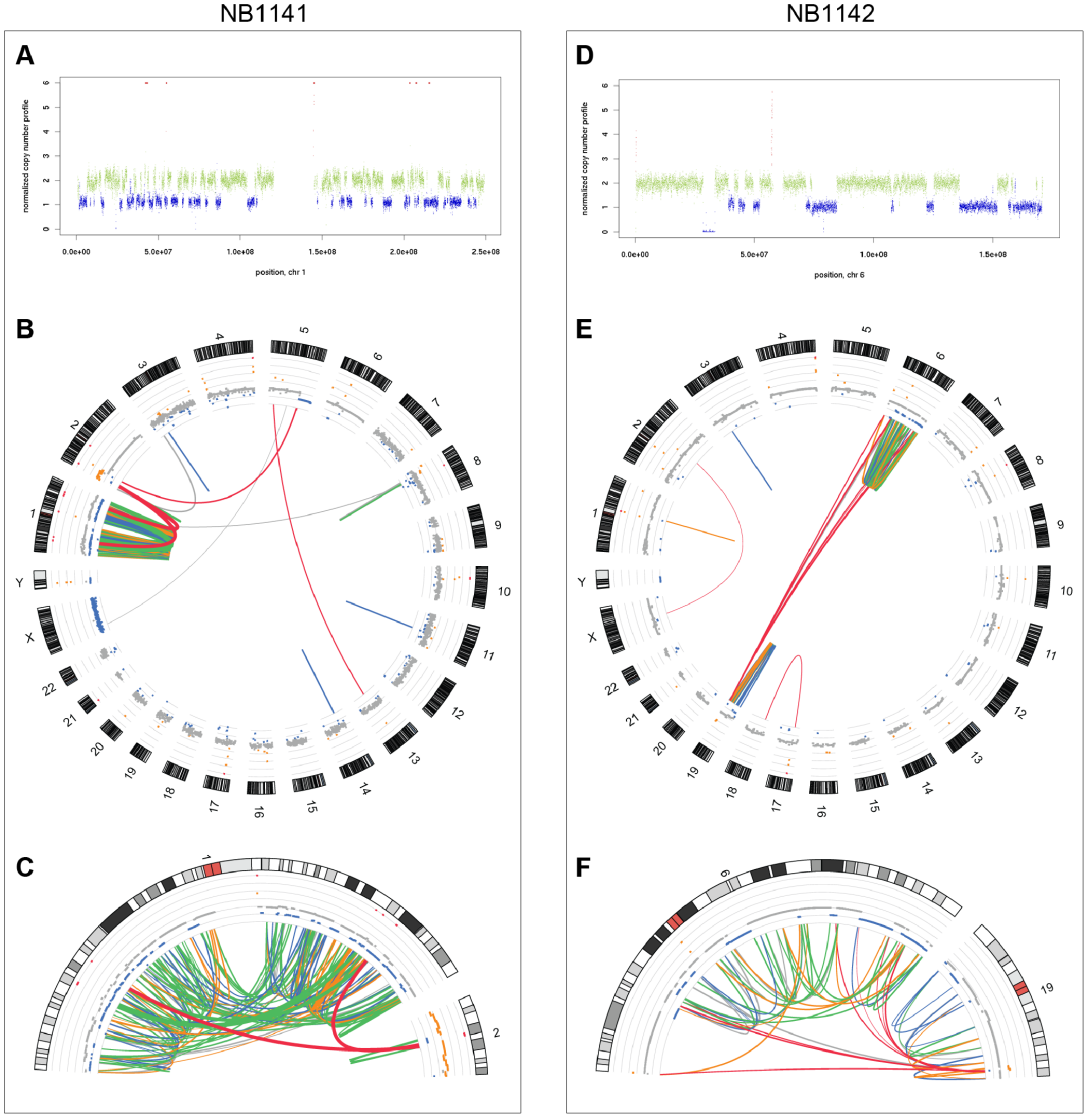
Chromothripsis in two primary NB tumors. FREEC analysis showing the shattering of: **A**, chromosome 1 in NB1141; **D**, chromosome 6 in NB1142. All inter-chromosomal rearrangements and intra-chromosomal SVs encompassing regions longer than 50 kb identified in mate-pair sequencing are visualized using the Circos tool. **B**, NB1141 tumor; **E**, NB1142 tumor; **C**, zoom on chromosome 1 and distal part of chromosome 2p arm (0–35 Mb) in NB1141; **F**, zoom on chromosomes 6 and 19 in NB1142. Line’s thickness is related to the numbers of pairs identified in each link.

We could unambiguously document chromothripsis in these two tumors. For NB1141, mate-pair analysis confirmed: (1) the shattering of chromosome 1, with copy number changes alternating between only two states, i.e. one or two copies (except for the presence of a few amplicons, Figure 3A); (2) the geographic localization of the rearrangements within only one chromosome (Figure 3B) and (3) the diversity of rearrangements that are observed, including PEM signatures for deletions, inversions and large duplications (Figure 3C and Table 2). A few inter-chromosomal rearrangements were also detected between different chromosomes (Figure 3B). In particular, rearrangements involving chromosomes 1 and 2 were documented by a very high number of pairs and actually involved six regions of amplification on chromosome arms 1p, 1q and 2p24. Figure S4 shows the potential structure of these amplicons. For NB1142, mate-pair analysis confirmed high level of rearrangements on chromosomes 6 and 19 (Figure 3E). We observed both inter and intra-chromosomal rearrangements targeting these two chromosomes (Figure 3F). Here again, segments within the rearranged chromosomes exhibited mainly copy number of one or two (Figure 3D, E) while all other chromosomes were present in the normal copy number of two.

For NB1141, we experimentally validated all the twelve intra-chromosomal SVs that were selected with PEM signatures corresponding to 4 deletions, 4 inversions and 4 large duplications (Table S4). For NB1142, 5 out of 6 selected inter-chromosomal rearrangements involving chromosomes 6 and 19 were validated (Table S4). None of the rearrangements were detected in the corresponding germline DNA. Breakpoint regions of these SV were sequenced at the base pair level.

For both primary tumors, we identified many SVs of short size (SV insert size <50 kb) (Table S2 and Table S3). Similarly to the cell lines, these short SVs were mostly deletions (33%) and insertions (36%) and they fell frequently in intergenic regions (64%) and intronic regions (31%). A large number of all predicted short SVs (53%) were annotated as known SVs in DGV [21].

### Expected Consequences of SVs on the Gene Structure

We classified links into various categories (see Methods) according to the expected consequences of the SV on the gene structure (Table 2). The damaging class of SVs includes the “truncated”, “possible chimera” and “may change function” classes. The non-damaging class comprises the “intergenic”, “promoter” and “does not change function” categories. A subset of SVs was classified as “may not change function”. For inter-chromosomal links, 29 to 71% of them were classified as damaging whereas for large intra-chromosomal SVs, this category represented 37 to 46% of the predicted SVs (Table 2). For short intra-chromosomal SVs, the percentage of damaging events was much smaller (2% to 9%, Table S3). This corresponded to 44, 109, 88 and 29 genes potentially damaged by the SVs in CLB-Ga, CLB-Re, NB1141 and NB1142, respectively. Among them, 12 genes were present in the Cancer Census List (n = 1 in CLB-Ga, n = 8 in CLB-Re, and n = 3 in NB1141) (http://www.sanger.ac.uk/genetics/CGP/Census/). None of these 12 genes was found recurrently altered by a “damaging” SV in several samples.

### Breakpoint Analysis Reveals a Subset of Rearrangements with Templated Insertion of Fragments of Nearby Sequences and Frequent Microhomology

**Figure 4:**
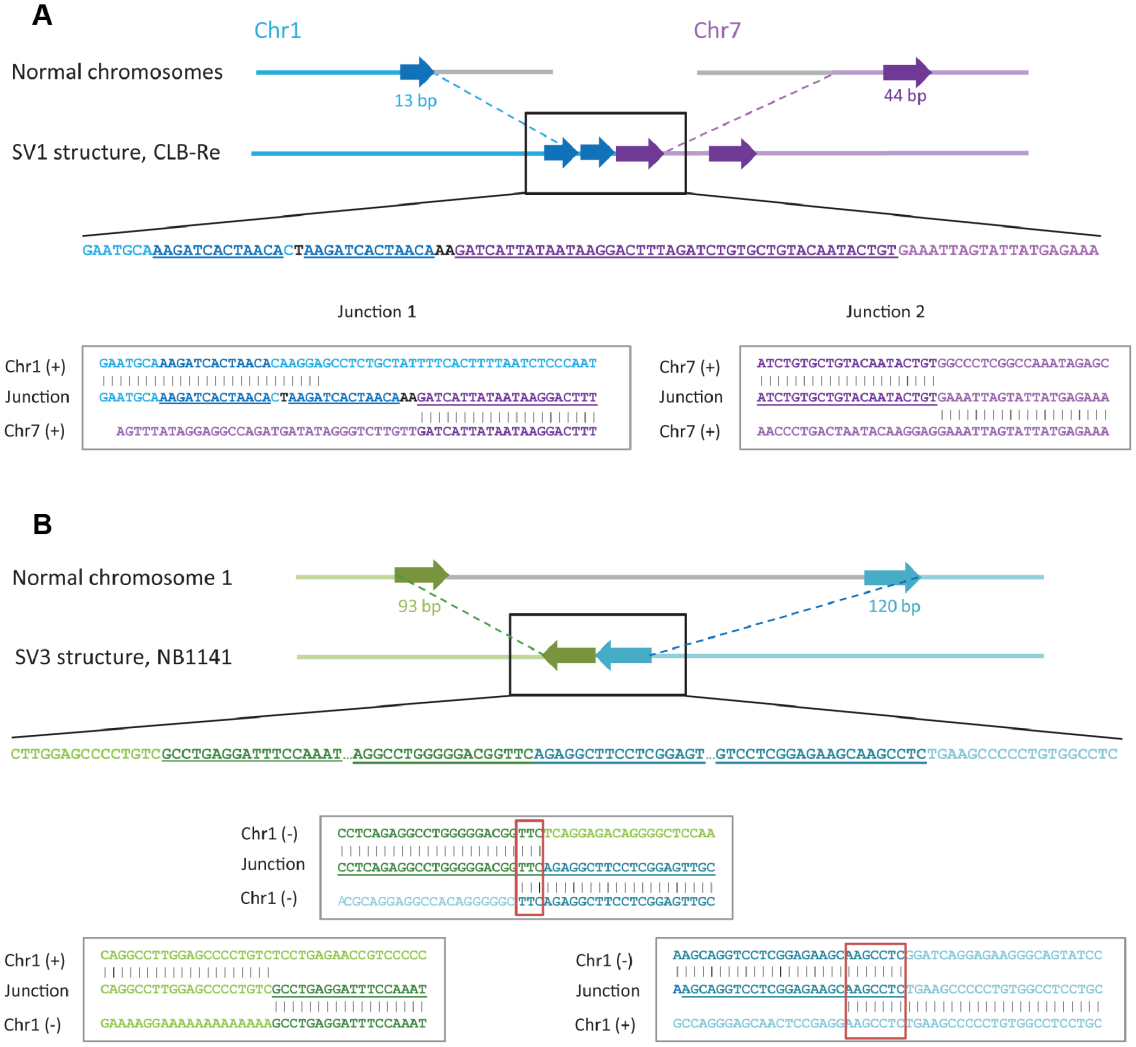
Examples of rearrangements with templated insertion of nearby sequences. **A**, Rearrangement SV1 between chromosomes 1 and 7 in CLB-Re; **B**, intra-chromosomal rearrangement SV3 on chromosome 1 in NB1141. In each panel, the top graph shows the schematic representation of normal chromosomes. Below is the schematic diagram of the SV. The nucleotide sequence of the rearrangement region (black box) is indicated below. Alignments in boxes illustrate the structure of each breakpoint within the rearrangement. Each partner in the rearrangement is shown by a different color. The parts of chromosomes that were lost during rearrangement are shown in grey. Regions that were duplicated (**A**) or inverted (**B**) are shown by arrows of a darker color in the diagram; corresponding sequences are underlined.

**Figure 5:**
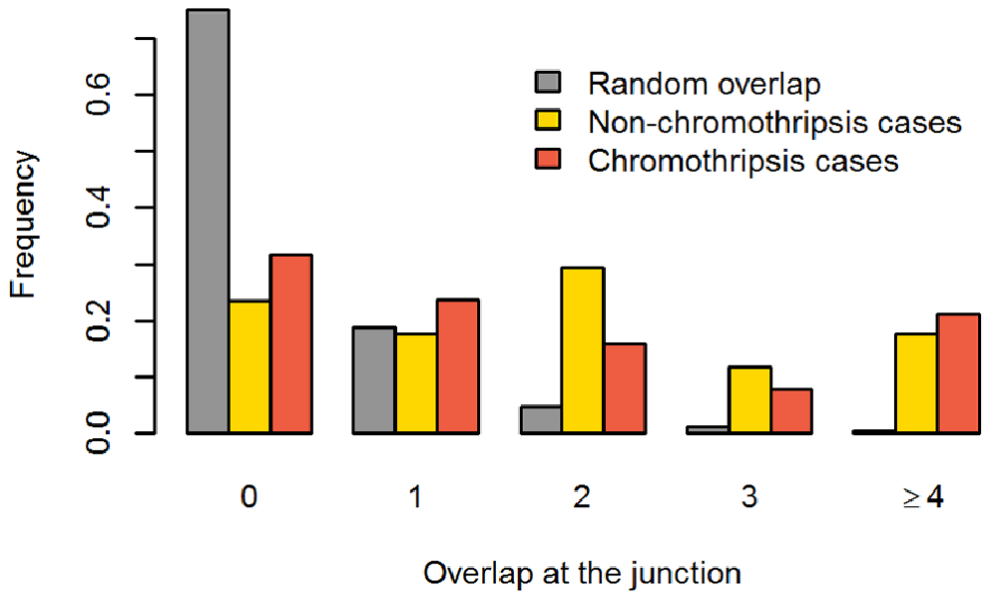
Microhomology at breakpoint junctions is found more frequently than expected by chance in chromothripsis and non-chromothripsis associated breakpoints. The histogram shows theoretical (grey) and observed frequencies of microhomology on validated breakpoint junctions for chromothripsis (orange) and non-chromothripsis cases (yellow).

For SVs validated by PCR, further Sanger sequencing of the fragments allowed characterization of the junctions at the base pair level. This analysis revealed more complex structures than expected with two or more breakpoints per rearrangement in 6 cases out of 51. In total, we characterized 59 DNA junctions (Table 3, Figure S5). In two cases, a genomic shard, i.e., a fragment derived from a known position of the human genome but located at some distance from the two joined fragments, was inserted at the junction between the two fragments. In four cases, we observed complex rearrangements with templated insertion of fragments of nearby sequences at the expected breakpoint (two cases are shown on Figure 4A and 4B; see also Table S4). Sequences at rearrangement junctions frequently exhibited microhomology (1 to 28 bp, Table S5). Indeed, such microhomology was observed in 40 out of 59 junctions characterized in the 4 samples (Table 3, Figure 5). In the same samples, we counted 12 cases of blunt ends. The number of junctions with microhomology was significantly higher than expected by chance in both non-chromothripsis (chi-square test p-value = 1.4e-36) and chromothripsis cases (chi-square test p-value = 4.3e-96). In 6 cases, junctions contained a short insertion of 1 to 17 bp (Table 3 and Table S4).

**Table 3:** Characteristics of junctions defined at the base pair level.

	CLB-Ga	CLB-Re	NB1141	NB1142
Nb of rearrangements analyzed at the bp resolution	11	23	12	5
Simple rearrangements	10	21	11	3
Complex rearrangements				
• with genomic shard	1 (SV7)	1 (SV15)		
• with templated insertion of fragments of nearby sequences		1 (SV1)	1 (SV3)	2 (SV1/SV5)
Nb of junctions	12	26	14	7
Pattern at junction				
• Micro-homology	10	15	9	6
• Blunt	2	6	4	–
• Insertions	–	5	1	1

We also searched for specific motifs potentially implicated in rearrangement formation within all characterized sequence junctions (Table S6) either at immediate or short proximity of the breakpoints (40 and 250 bp of genomic sequence surrounding the breakpoint, respectively). For cell lines, this analysis revealed an enrichment of one S/MAR (Scaffold/matrix attachment regions) motif in CLB-Re at immediate proximity. In primary tumors exhibiting chromothripsis, two motifs were significantly over-represented in NB1141 but we could not demonstrate an enrichment of the same motifs in NB1142 (Table S6).

### RNA-seq Analysis Allows the Identification of Abnormal Transcripts Expressed from Genomic Rearrangements

In order to determine which SVs predicted to modify normal gene structure indeed resulted in abnormal RNA products, we performed RNA-seq for the four cases. After mapping paired-end reads to the reference genome as well as to putative exon-exon junctions, we checked whether we can find clusters of pairs corresponding to the predicted SVs. Here, we considered only SV possibly resulting in chimeric transcripts or in transcripts with deleted/duplicated/inverted exons. We annotated an SV as “expressed” if there was more than one read pair linking the exons or, in some cases, introns corresponding to the SV.

Among 86 SVs that could have led to abnormal RNA transcripts (chimeras or transcripts with violation of exon structure), only sixteen (19%) produced such transcripts at the level detectable with the RNA-seq experiment (Table 4). In 24 cases, genes involved in the SV were not expressed in our samples, and so the abnormal transcripts.

**Table 4:** Abnormal transcripts confirmed with RNA-seq.

Genes	Type	# RNA-seqread pairs	Sample	Comment
PTPRD	deletion	13	CLB-Ga	Deletion of two exons
PDGFRB///LRRC4C	chimera or truncated	11	CLB-Ga	5′ PDGFRB ->3′ LRRC4C (2 pairs); 5′ PDGFRB -> intron LRRC4C (8 pairs); intron PDGFRB -> intron LRRC4C (1 pair)
ALK///FHIT	chimera	21	CLB-Re	5′ ALK ->3′ FHIT
CRIM1///THADA	chimera	5	CLB-Re	5′ CRIM1 -> intron of FHIT ->3′ THADA
THADA///CADPS	chimera or truncated	9	CLB-Re	5′ THADA -> intron CADPS
XRCC3///ODZ4	chimera or truncated	8	CLB-Re	5′ XRCC3 ->3′ ODZ4 (1 pair); 5′ XRCC3 -> intron ODZ4 (7 pairs)
ATL2///MYT1L	chimera	21	CLB-Re	5′ ATL2 ->3′ MYT1L
THADA///ARHGEF33	chimera or truncated	39	CLB-Re	5′ THADA ->3′ ARHGEF33 (19 pairs); 5′ THADA -> intron ARHGEF33 -> exon ARHGEF33 (20 pairs)
ADAMTS9///C3orf67	chimera	59	CLB-Re	5′ ADAMTS9 ->3′ C3orf67
IRF2///MLF1IP	chimera	41	CLB-Re	5′ IRF2 ->3′ MLF1IP
NBAS///KCNK2	chimera (amplicon)	72	NB1141	5′ NBAS ->3′ KCNK2 (64 pairs); intron NBAS -> intron KCNK2 (8 pairs)
KAZN///KLHL12	possibly not functional	3	NB1141	intron KAZN ->3′ KLHL12
CAPN2///TSSK3	chimera	3	NB1141	5′ CAPN2 ->3′ TSSK3
GCLM///NFIA	chimera	3	NB1141	5′ GCLM ->3′ NFIA
RABGAP1L///NEXN	chimera	2	NB1141	5′ RABGAP1L ->3′ NEXN
NCOA7///EYS	chimera	10	NB1142	5′ NCOA7 ->3′ EYS

“5′ GeneX ->3′ GeneY”: a transcript starts at the promoter of GeneX and finishes at the transcription end of the GeneY, no intronic shards included at the translocation; “5′ GeneX -> intron of GeneY ->3′ GeneZ”: an intron from GeneY is included in the transcript starting from the promoter of GeneX and finishing at the transcription end of GeneZ; “5′ GeneX -> intron GeneY”: a transcript starts at the promoter of GeneX, includes a part of an intron of GeneY, there is no read pairs showing that exons of GeneY are included into the abnormal transcript.

Several expressed chimeras involved genes that had been shown to be frequently mutated or targeted by rearrangements in NB. This list includes *ALK*, as well as *NBAS*, *PTPRD* and *ODZ4* [23]. Interestingly, we found Rho guanine nucleotide exchange factor (GEF) 33 (*ARHGEF33*) involved in a chimeric transcript. In the recent work by Molenaar and colleagues [23], another Rho GEF, *ARHGEF12*, also showed a translocation of an unknown function.

## Discussion

In this study, we used a mate-pair strategy combined to RNA-seq to characterize in-depth genomic rearrangements in NB cell lines and primary tumors. The two cell lines present with typical genetic alterations of NB, *i.e.*, unbalanced translocations, and one of them also exhibits *MYCN* amplification. The two tumors belong to the group of NB exhibiting a profile of chromothripsis that has been described in a variety of cancers [17], [24]–[26] and recently shown to occur in 18% of high-stage NB [23]. Altogether, these cases are undoubtedly representative of a quite large proportion of NB.

Mate-pair sequencing, since dealing with large DNA fragments, is a rather efficient way to identify large rearrangements. In our experiments, we had very low sequence coverage (<1X, relative to the six billion-base pair diploid genome). Nevertheless, since we used 3–4 kb fragments, we obtained decent effective coverage (from 8X to 14X), meaning that we expected to have about 8–14 mate pairs per SV. For the CLB-Ga cell line our procedure was indeed efficient to characterize SVs associated with copy number changes since it allowed the precise identification of 17 out of 20 breakpoints associated with copy number changes detected by FREEC. In the CLB-Re cell line, the efficiency of the mate-pair strategy to identify SVs associated with copy number changes was not as high as in the CLB-Ga cell line. This lower efficiency may rely on the lower effective coverage obtained for the CLB-Re sample (8X) compared to that of the CLB-Ga sample (10X).

Our mate-pair analysis identified 269 genes with function potentially damaged by SVs in the 4 analyzed samples. Among them, 12 were present in the Cancer Gene Census (Release 2012-03-15) [27]. The rearrangements of the *PDGFRB*, *ATIC*, *EML4*, *FOXP1*, *MYCN* and *ABL2* genes were predicted to result in truncated forms of the encoded products. Using RNA-seq, we were able to see expression of truncated forms for *PDGFRB*, *FOXP1* and *ABL2* (data not shown). Although it is unknown whether expression of these truncated forms can have functional consequences in NB, short isoforms of *FOXP1* have been shown to act like oncogenes in diffuse large B-cell lymphoma and in mucosa-associated lymphoid tissue lymphoma [28]. Interestingly, except for the *MYCN* gene which is targeted by amplification in cancer and for *FOXP1*, the other SVs in these genes previously described in cancer samples were causing gene fusions and not truncated forms of the proteins. Four genes including *ALK* at 2p23.2, *FHIT* at 3p14.2, *MITF* at 3p13 and *TPR* at 1q25 were predicted to be affected by several different SVs. Rearrangements targeting the *ALK* gene in the CLB-Re have also been explored using a capture and paired-end sequencing strategy [29]. Results of both approaches were fully consistent. However, since this cell line is tetraploid [14], it is difficult to figure out whether the different SVs target the same allele and what are the functional consequences of such rearrangements. We also discovered abnormal transcripts that included genes recently described as being frequently altered in NB, i.e. *PTPRD* and *ODZ4* [23] and involved in neuritogenesis. We did not detect any SV in the chromatin-remodeling genes *ARID1A* and *ARID1B* that have been shown recently to be targeted by deletions in around 10% of NB tumors [30].

We next examined whether identified SVs targeted genes located within common fragile sites that have been associated with DNA instability in cancer cells. The *FHIT* gene which is targeted by multiple rearrangements in the CLB-Re sample overlies the fragile site *FRA3B* [31] whereas the *WWOX* gene overlapping the *FRA16D* site [32] is implicated in the CLB-Ga cell line with two rearrangements. We noticed that the rearrangement between the amplified regions at 1q41 and 2p24.3 (that includes the *MYCN* oncogene) in NB1141 results in a chimera between the *KCNK2* and *NBAS* genes. Interestingly, it has been shown recently that *MYCN* amplicon borders frequently cluster in the *FRA2C* region, consisting of *FRA2Ctel* at 2p24.3 that contains the *NBAS* gene and *FRA2Ccen* at 2p24.2 [33]. The *NBAS* gene was also found to be targeted by three different SVs in the CLB-Re sample. This observation is consistent with previously reported results showing that this gene was targeted by multiple events in several NB cases with *MYCN* amplification [34].

The mechanisms leading to genomic instability in NB remain poorly understood. Defects in DNA maintenance or repair pathways may be responsible for SVs. Interestingly, the Break-Induced Replication (BIR) mechanism, has been shown to lead to the accumulation of unbalanced translocations in the yeast *Saccharomyces cerevisiae* [35]. Regarding the phenomenon of chromothripsis, several potential non-exclusive mechanisms have now been proposed. The first report suggested that it may rely on the fragmentation of a chromosome with massive DNA double strand breaks followed by non-homologous end joining (NHEJ)-mediated repair of the resulting fragments. The highly localized nature of the breaks affecting a specific chromosome or chromosome arm may be linked to the spatial organization of chromosomes and particularly to their compaction during mitosis [17]. Aberrant DNA replication resulting in fork stalling and template switching (FoSTeS) and/or microhomology-mediated Break-Induced Replication (MMBIR) may also serve as a basis for chromothripsis [36]. The analysis of SVs breakpoint features in NB may provide interesting clues in the understanding of the mechanisms involved in genomic rearrangements. We first investigated a series of 48 motifs potentially implicated in rearrangement formation and also analyzed the GC, polypurine and polypyrimidine contents as well as the alternating purine-pyrimidine motifs in sequences surrounding breakpoints. We did not detect significant enrichment. The contribution of known particular motifs in the local environment of the breakpoints to the precise location of breaks and subsequent repair seems therefore modest in the analyzed samples. However, the analysis of 59 breakpoints characterized to the base pair level in the present study revealed frequent microhomology in up to 68% of the junctions, in both non-chromothrispsis and chromothrispsis cases. Frequent short microhomology tracts at the breakpoint junctions have also been reported in somatic rearrangements of other cancers including for instance breast cancer [17], colorectal cancer [24] and medulloblastoma [26]. Moreover, microhomology was reported at the amplicon junctions in NB cases exhibiting *MYCN* amplification [16]. In the two NB primary tumors with chromothripsis as well as in the CLB-Re cell line, we characterized several rearrangements with templated insertion of fragments of nearby sequences. However, no major difference in the characteristics of the junctions was noticed between SVs occurring in the presence or absence of the chromothripsis context. Altogether, our data and data from the literature therefore supports the hypothesis that not only NHEJ-mediated repair but also replicative processes such as Break-Induced Replication (BIR), FoSTeS and/or MMBIR may account for genomic rearrangements in NB, as it has been suggested in other cancers from adult or children.

## Materials and Methods

### Ethics Statement

Regarding the analysis of human samples, this study was authorized by the decision of the ethics committee “Comité de Protection des Personnes Sud-Est IV”, reference L07–95 and L12–171. Written informed consent for the study was obtained from parents according to national law. The ethics committee of the IRCIV (Institut de Recherche en Cancérologie Intégrée de Villejuif), registered as number 26 to the National Committee (Comité National de Réflexion Ethique sur l’Expérimentation Animale) approved the mouse xenograft analysis (protocol number 2010-02).

### NB Samples

Two NB cell lines, CLB-Ga and CLB-Re (Table 1), previously characterized by 24-color karyotyping [14], [20], FISH [37], and array-CGH [13] were analyzed by mate-pair sequencing. A lymphoblastoid cell line, LL-Ga, derived from the lymphocytes of the patient from which the CLB-Ga cell line was established was also studied by mate-pair sequencing and used as a control. Furthermore, we analyzed genomic DNA from a bone marrow sample of the Ga patient known to be contaminated with tumor cells. For the Re patient, we obtained DNA from tumor cells at diagnosis, as well as DNA from blood. Whole-genome amplification (WGA) was performed on these samples to further confirm rearrangements. Two NB primary tumors, NB1141 and NB1142, corresponding to a stage 3 and stage 2 NB, respectively, according to the INSS classification [38] were also subjected to mate-pair analysis. For both tumors, native tumor DNA was used for mate-pair sequencing; however, these DNAs as well as the matched constitutional DNAs were subjected to WGA before validation of putative SVs by PCR. For RNA-seq, total RNA was extracted from CLB-Ga and CLB-Re samples and from NB1142 tumor. For NB1141, we extracted RNA from a mouse xenograft of the primary tumor of the patient. Array-CGH indeed indicated that the genomic profiles of both tumors were highly similar (Figure S6). A general workflow of this study is provided in Figure S7.

### Mate-pair Sequencing

The DNA libraries were prepared following Illumina protocol “Preparing 2–5 kb Samples for Mate Pair Library Sequencing” using 10 micrograms of genomic DNA (see Methods S1 for the protocol description). We used standard specifications (initial gel size selection: around 3 kb; adapter-ligated library amplification: 18 PCR cycles; final size selection: 400–600 bp). The Illumina Genome Analyzer IIx system generated paired-end sequences of 35, 50 or 76 nucleotides. Images from the instrument were processed using the manufacturer’s software to generate FASTQ sequence files. The sequencing resulted in about 70 million raw paired reads per sample (Table S1).

### RNA Sequencing

PolyA mRNAs were purified from 10 µg of total RNA for the CLB-Ga, CLB-Re and NB1141 samples (3 µg for NB1142) using the Purification of poly(A) RNA NucleoTrap® mRNA kit according to the manufacturer’s protocol (Macherey Nagel, Düren, Germany). We prepared RNA-seq SOLiD libraries following the SOLiD® Total RNA-Seq Kit protocol using 100 ng of polyA mRNAs for the CLB-Ga, CLB-Re and NB1141 samples (22 ng for NB1142) (see Methods S1 for the protocol description).

The SOLiD V4 and 5500 systems generated paired-end sequences of 50 and 35 nucleotides for 5′ and 3′ ends, respectively. Multiplex samples were run on two flowchips of the SOLiD V4 and one flowcell (6 lanes) of the 5500 SOLiD system. Images from the instrument were processed using the manufacturer’s SOLiD V4 ICS v4.0.2 and 5500 Series Genetic Analyser ICS v1.2.1 software to generate sequence CSFASTA and quality QUAL files. The sequencing resulted in about 400 million raw paired reads per sample (Table S1).

### Data Alignment

#### Whole genome

For each sample, reads were aligned to the human NCBI Build 37 reference genome (hg19, downloaded from http://genome.ucsc.edu) using Bowtie [39] and BFAST [40]. Any potential PCR duplicates and singletons (read pairs mapping in inward orientation and with an abnormally short insert size) were excluded (see Methods S1 for the detailed workflow). Finally, we obtained from 906 to 2 155 thousand “abnormal” read pairs and from 13 to 30 million “normal” read pairs per sample (Table S1).

#### RNA-seq

For each sample, reads were aligned to the human NCBI Build 37 reference genome (hg19) and a library of exon junctions provided by Lifescope using the SOLiD Lifescope v.2.5 software. Reads were filtered using mapping quality (threshold 5). Any potential PCR duplicates were excluded (see Methods S1). Finally, we obtained from 4 to 12 million read pairs per sample (Table S1).

### Annotation of Copy Number Status and Prediction of SVs

To detect copy-number alterations in our samples, we used FREEC v3.9 [19] on “normal” and uniquely mapped reads with filtered duplicates (Methods S1). We ran SVDetect [18] using “abnormal” reads of each sample and processed the output using a strategy presented in Methods S1 to predict structural variants (translocations, insertions, deletions, tandem duplications, etc.). A detailed workflow for detection of copy number alterations and structural variants using whole genome sequencing data is provided in Figure S8.

### Annotation of Predicted SVs

We annotated predicted SVs with gene information (RefSeq Release 50): For each putative breakpoint we checked whether it fell between the transcription start site (TSS) and transcription end of a gene. Also, we annotated each gene putatively disrupted by an SV with one of the following tags: “Truncated”, “Possible chimera”, “Does not change function”, “May change function”, “May not change function” (Figure S9, Methods S1).

### Detection of Expressed SVs

For SVs annotated as “Possible chimera” and “May change function” (Figure S2 and S9), we checked the presence of abnormal RNA transcripts. We considered an SV expressed if we detected at least two RNA-seq read pairs spanning the SV junction: The RNA-seq pair ends were allowed to map either to the intronic regions adjacent to the breakpoints or to the exons following the breakpoints.

### Whole-Genome Amplification (WGA)

WGA was performed on 10 ng of genomic DNA with the GenomiPhi V2 DNA Amplification Kit (GE Healthcare) according to manufacturer’s instructions.

### Validation of Predicted Rearrangements by PCR

Primers (available on request) were designed using Primer3 to span the possible breakpoint by locating them in a window of 1 kb outside the paired-end reads. PCR was performed with AmpliTaq Gold® (Applied Biosystems) or TaKaRa Taq™ Polymerase (EX or LA, Ozyme) on 20 ng of genomic DNA or WGA-DNA using a touch-down PCR program. Rearrangements giving a clear band in the tumor DNA with no matching band in the normal DNA were defined as somatic; all reactions were performed at least twice. PCR products were sequenced by conventional Sanger capillary sequencing methods and compared to the reference genome to identify breakpoints.

### Public Database Accession Numbers for Data

SRA accession number ERP001414 for mate pair sequencing data. ArrayExpress accession number E-MTAB-1367 for RNA-seq data (http://www.ebi.ac.uk/ena/data/view/ERP001988).

## Supporting Information

Figure S1 **Output of FREEC generating normalized copy number profiles for LL-Ga, CLB-Ga, CLB-Re, NB1141 and NB1142.**
(PDF)

Figure S2 **Rearrangements detected in the CLB-Re cell line established at relapse were already present at diagnosis.** A, A subset of the links identified by the mate-pair analysis between chromosomes 2 and 3 was further confirmed by PCR experiments, both on the genomic DNA of the CLB-Re cell line and also after whole genome amplification (WGA). DNA from the matched tumor at diagnosis as well as from lymphocytes was also investigated. B, Examples of rearrangements detected after WGA on the gDNA of the cell line established at relapse that were also detected after WGA of the DNA at diagnosis, demonstrating that these rearrangements were not relapse-specific and not linked to the cell culture process. No SV was detected in the germline DNA.
(PDF)

Figure S3 **Complex rearrangement between chromosomes 12 and 20 in CLB-Ga.** The copy number profile for chromosome 20 together with a link between chromosomes 12 and 20 (SV7) suggested an unbalanced t(12,20) translocation. With a resolution of 30 kb no copy number change were observed in the corresponding region of chromosome 12. Analysis of intra-chromosomal SVs on chromosome 12 followed by Sanger sequencing revealed a complex structure of SV11. Altogether, our analysis predicts a rearrangement including a fragment from chromosome 20 (17.4 Mb, magenta) and three fragments from chromosome 12 (30 kb, 243 bp and 1.8 Mb, purple, turquois and blue, respectively).
(PDF)

Figure S4 **Predicted amplicons’ structure in NB1141.** A, normalized copy number profiles for chromosomes 1 and 2 determined by the FREEC method. B, Scheme of the potential structure of two amplicons involving regions located on chromosomes 1 and 2. The upper part represents the normal position of the regions involved in the amplicons, whereas the middle and lower parts deciphers the organization of those regions within the two amplicons. The direction of the arrows indicates the orientation of each fragment. Amplicon A6 contains the *MYCN* gene.
(PDF)

Figure S5 **Junctions identified at the base pair level in CLB-Ga, CLB-Re, NB1141 and NB1142.**
(PDF)

Figure S6 **Copy number profiles for the primary tumor NB1141 (A) and xenografted tumor (B) obtained using Roche NimbleGene CGH microarrays.** Chromosome 1 of the NB1141 tumor has the signature of chromothripsis (Figure 3A–C). Here, we demonstrate that the xenografted tumor profile on chromosome 1 is identical to the one of the primary tumor with an exception for the amplicon structure A3-A1-A4 (see details in figure S4) which is not present in the xenografted tumor. Thus, the xenographed sample can be used for validation of expression of abnormal RNA transcripts resulting from the chromothripsis with RNA-seq.
(PDF)

Figure S7 **Data processing workflow for the four analyzed NB samples.**
(PDF)

Figure S8 **Analysis workflow for detection of copy number alterations and structural variants using whole genome sequencing data.**
(PDF)

Figure S9 **Annotation of predicted structural variants (SVs) with genomic information.** Gene exons are shown by large rectangles (dark blue and purple), introns are shown by thin arrows, 5′ and 3′ UTRs are shown by small rectangles (dark blue and purple). A read pair representative of the SV signature is shown by two connected rectangles (light blue) with white arrows showing the direction of sequencing. We say that a link falls in a gene if any of corresponding reads falls between the gene transcription start site (TSS) and transcription end. If reads fall within a region up to 2 kb upstream gene TSS, we annotate the corresponding end of the link as falling in a promoter region.
(PDF)

Figure S10 **Observed distribution of the insert sizes.**
(PDF)

Table S1 **Read statistics (total number of sequencing reads, aligned reads, insert size distibution, normal and abnormal reads).**
(PDF)

Table S2 **Structural variants predicted by SVDetect for 2 tumors and 3 cell lines.** See Methods S1 for filters applied.
(XLS)

Table S3 **Intra-chromosomal rearrangements predicted by SVDetect with insert size <50 kb.**
(PDF)

Table S4 **Validated structural variants (SVs) analyzed at the base pair level.**
(XLS)

Table S5 **Theoretical and observed frequencies of microhomology on validated breakpoint junctions in chromothripsis and non-chromothripsis cases.**
(PDF)

Table S6 **Statistically significant enrichment of motifs at rearrangement breakpoints.**
(XLS)

Methods S1. (PDF)
