# Supplementary material for: Breakpoint Features of Genomic Rearrangements in Neuroblastoma with Unbalanced Translocations and Chromothripsis
Source: PLoS One. 2013 Aug 26;8(8):e72182. doi: 10.1371/journal.pone.0072182 (PMC3753337; doi:10.1371/journal.pone.0072182)
Supplement: Figure S1 — Output of FREEC generating normalized copy number profiles for LL-Ga, CLB-Ga, CLB-Re, NB1141 and NB1142. (PDF) [file pone.0072182.s001.pdf]

Supplementary figure S1: Output of FREEC generating normalized copy number profiles for LL-Ga, CLB-Ga, CLB-Re, NB1141 and NB1142.

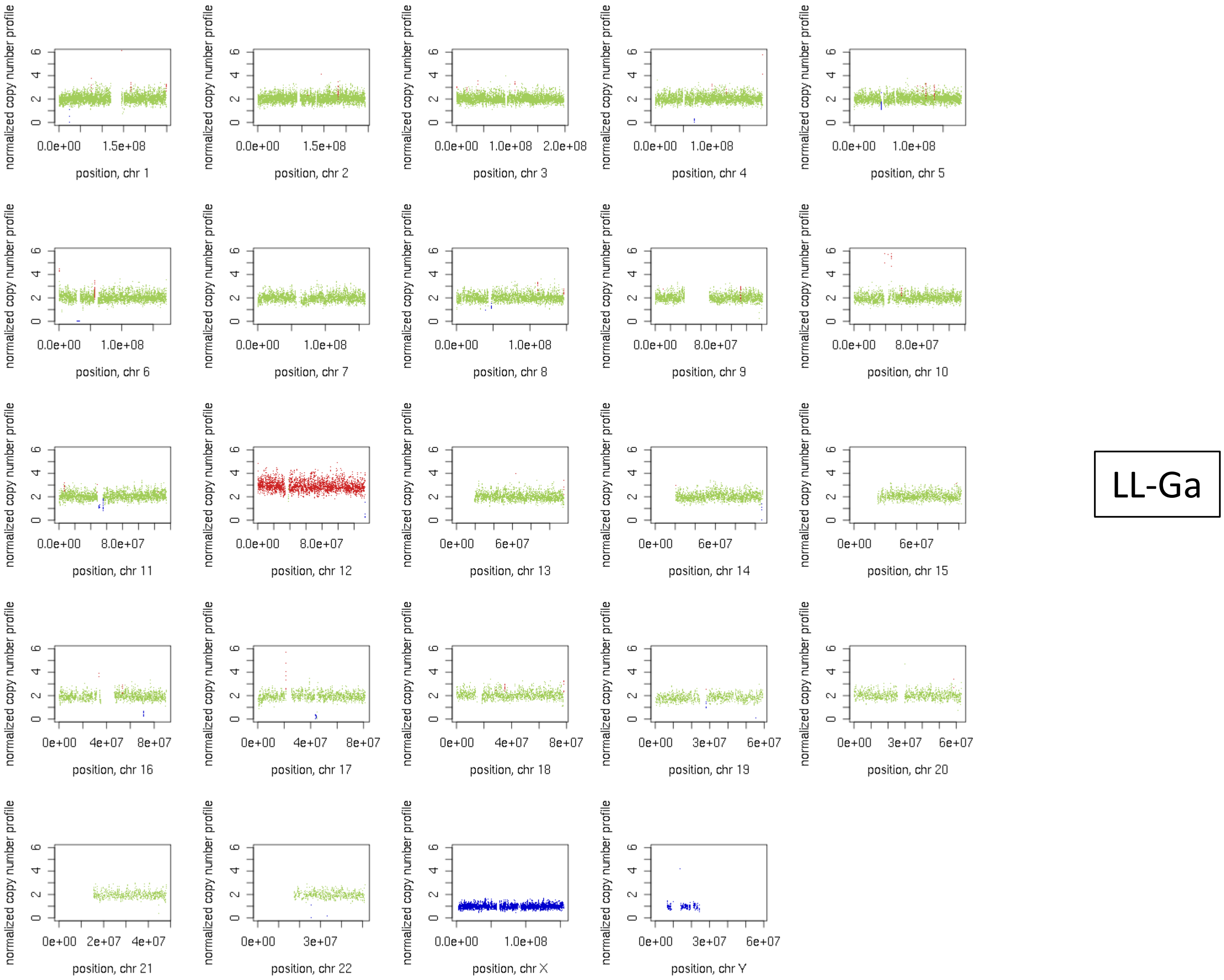

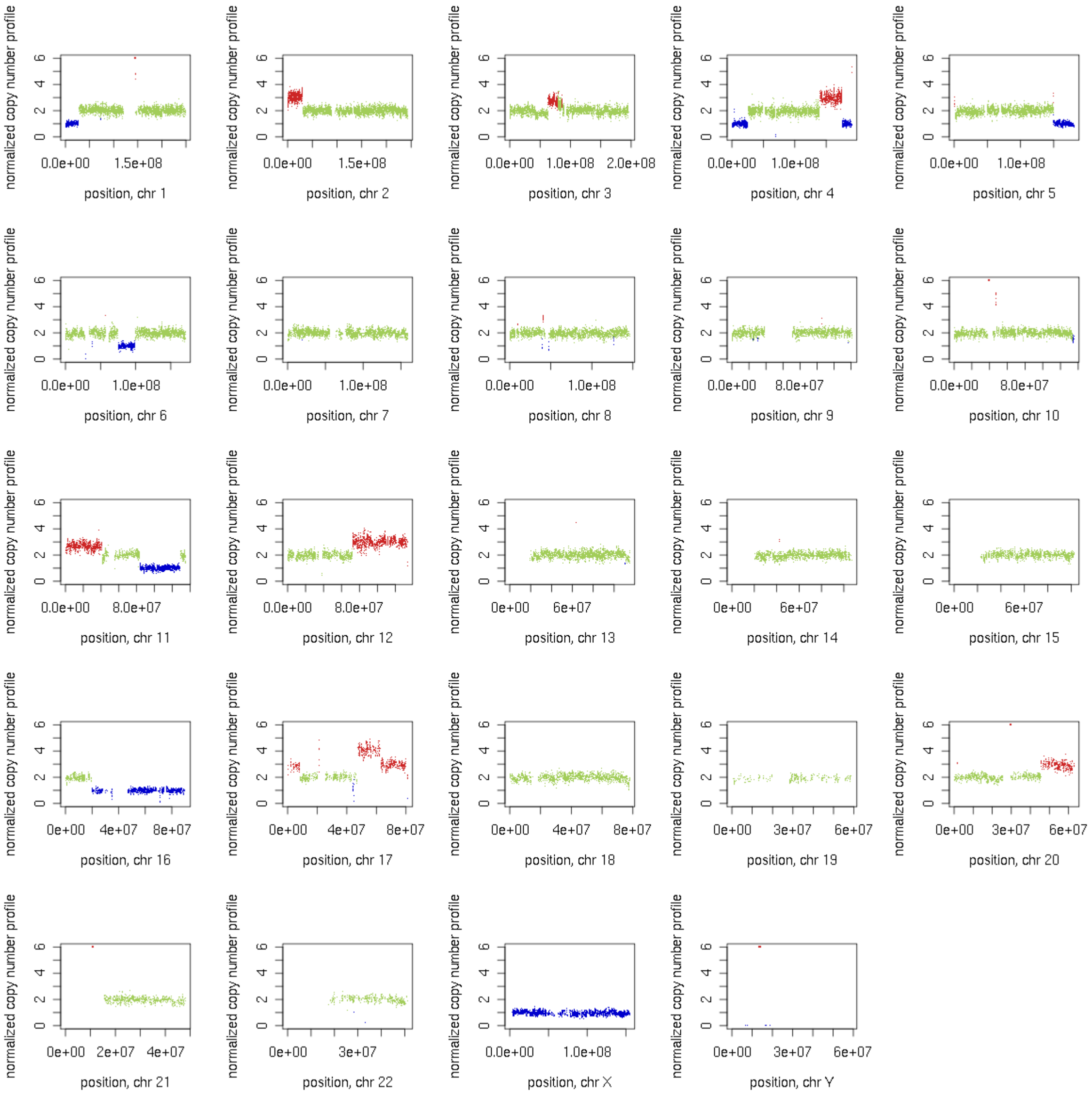
normalized copy number profile
position, chr 1
position, chr 2
position, chr 3
position, chr 4
position, chr 5
position, chr 6
position, chr 7
position, chr 8
position, chr 9
position, chr 10
position, chr 11
position, chr 12
position, chr 13
position, chr 14
position, chr 15
position, chr 16
position, chr 17
position, chr 18
position, chr 19
position, chr 20
position, chr 21
position, chr 22
position, chr X
position, chr Y

CLB-Ga

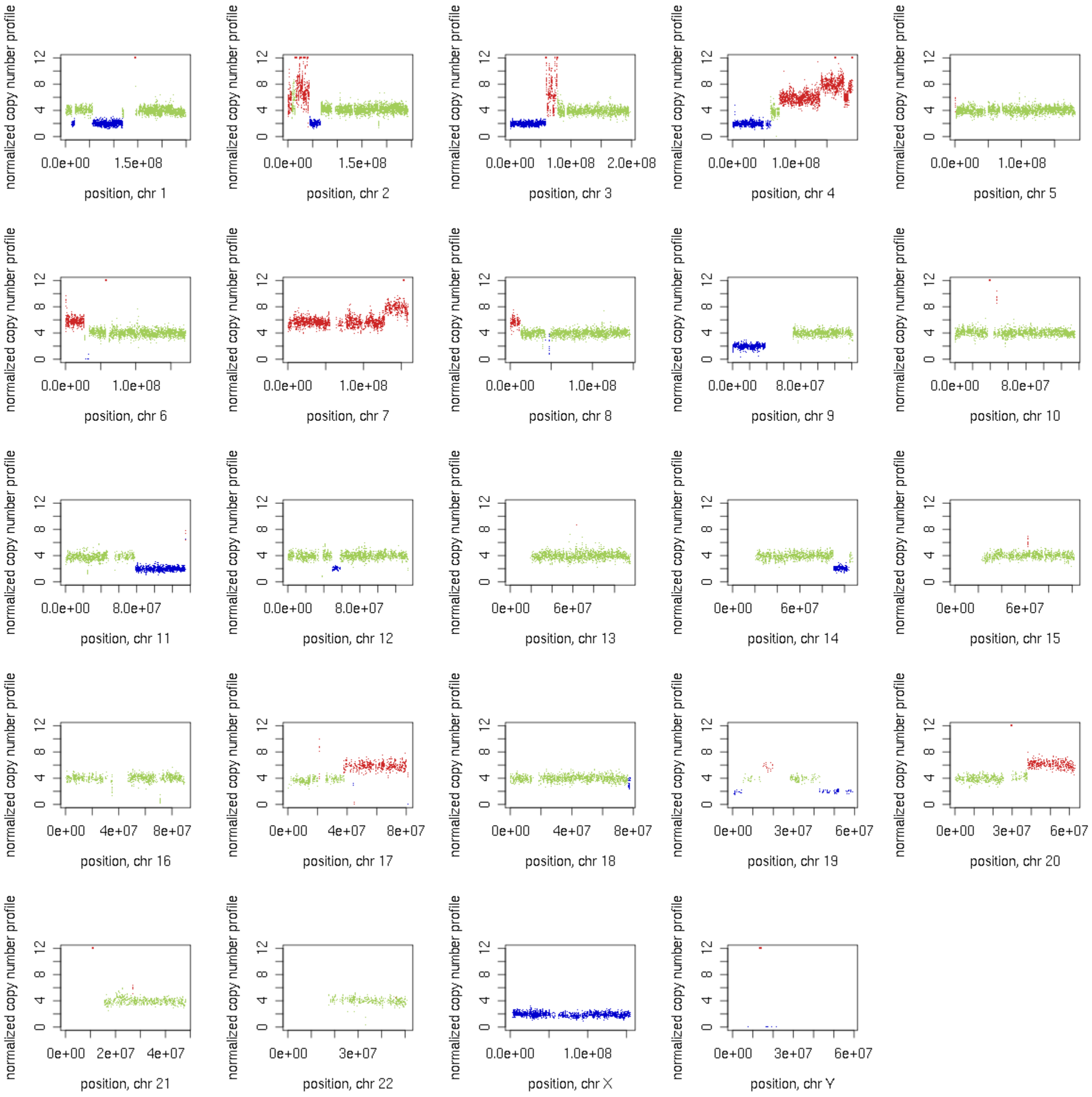
normalized copy number profile
position, chr 1
position, chr 2
position, chr 3
position, chr 4
position, chr 5
position, chr 6
position, chr 7
position, chr 8
position, chr 9
position, chr 10
position, chr 11
position, chr 12
position, chr 13
position, chr 14
position, chr 15
position, chr 16
position, chr 17
position, chr 18
position, chr 19
position, chr 20
position, chr 21
position, chr 22
position, chr X
position, chr Y

CLB-Re

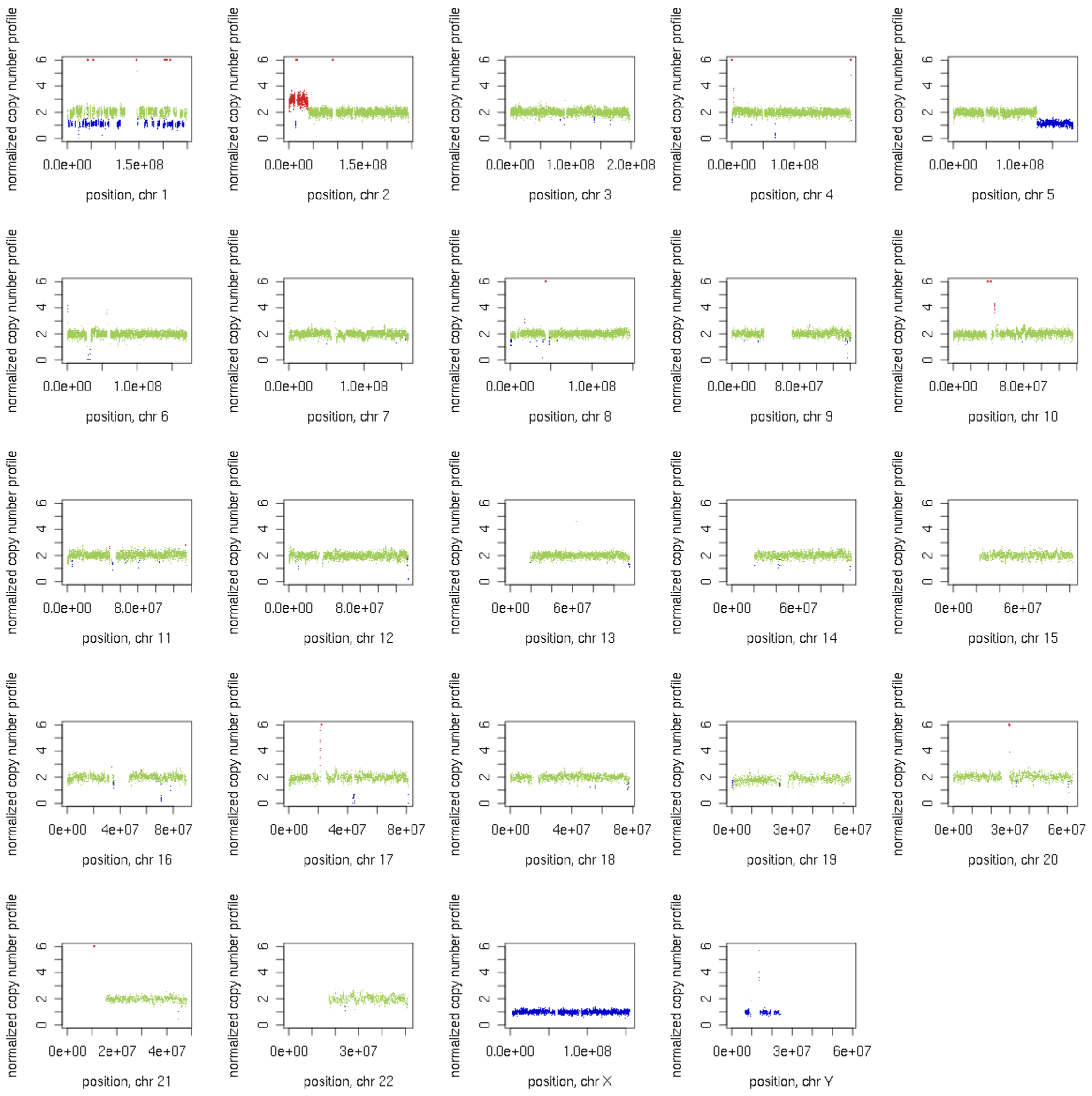
normalized copy number profile
position, chr 1
position, chr 2
position, chr 3
position, chr 4
position, chr 5
position, chr 6
position, chr 7
position, chr 8
position, chr 9
position, chr 10
position, chr 11
position, chr 12
position, chr 13
position, chr 14
position, chr 15
position, chr 16
position, chr 17
position, chr 18
position, chr 19
position, chr 20
position, chr 21
position, chr 22
position, chr X
position, chr Y

NB1141

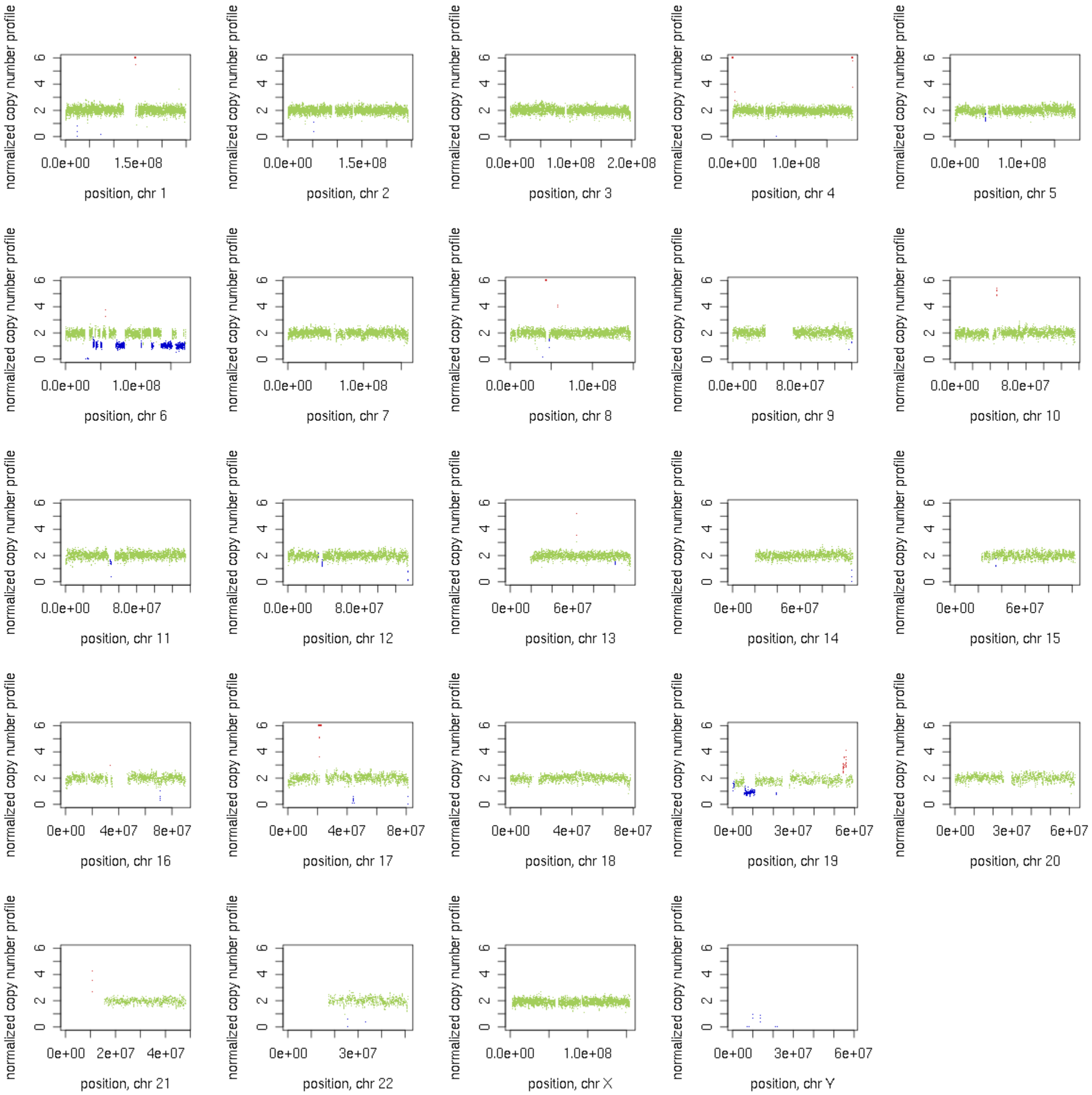
normalized copy number profile
position, chr 1
position, chr 2
position, chr 3
position, chr 4
position, chr 5
position, chr 6
position, chr 7
position, chr 8
position, chr 9
position, chr 10
position, chr 11
position, chr 12
position, chr 13
position, chr 14
position, chr 15
position, chr 16
position, chr 17
position, chr 18
position, chr 19
position, chr 20
position, chr 21
position, chr 22
position, chr X
position, chr Y

NB1142
