# Supplementary material for: Breakpoint Features of Genomic Rearrangements in Neuroblastoma with Unbalanced Translocations and Chromothripsis
Source: PLoS One. 2013 Aug 26;8(8):e72182. doi: 10.1371/journal.pone.0072182 (PMC3753337; doi:10.1371/journal.pone.0072182)
Supplement: Figure S2 — Rearrangements detected in the CLB-Re cell line established at relapse were already present at diagnosis. A, A subset of the links identified by the mate-pair analysis between chromosomes 2 and 3 was further confirmed by PCR experiments, both on the genomic DNA of the CLB-Re cell line and also after whole genome amplification (WGA). DNA from the matched tumor at diagnosis as well as from lymphocytes was also investigated. B, Examples of rearrangements detected after WGA on the gDNA of the cell line established at relapse that were also detected after WGA of the DNA at diagnosis, demonstrating that these rearrangements were not relapse-specific and not linked to the cell culture process. No SV was detected in the germline DNA. (PDF) [file pone.0072182.s002.pdf]

**Supplementary figure S2**: Rearrangements detected in the CLB-Re cell line established at relapse were already present at diagnosis. A, A subset of the links identified by the mate-pair analysis between chromosomes 2 and 3 was further confirmed by PCR experiments, both on the genomic DNA of the CLB-Re cell line and also after whole genome amplification (WGA). DNA from the matched tumor at diagnosis as well as from lymphocytes was also investigated. B, Examples of rearrangements detected after WGA on the gDNA of the cell line established at relapse that were also detected after WGA of the DNA at diagnosis, demonstrating that these rearrangements were not relapse-specific and not linked to the cell culture process. No SV was detected in the germline DNA.

**A**

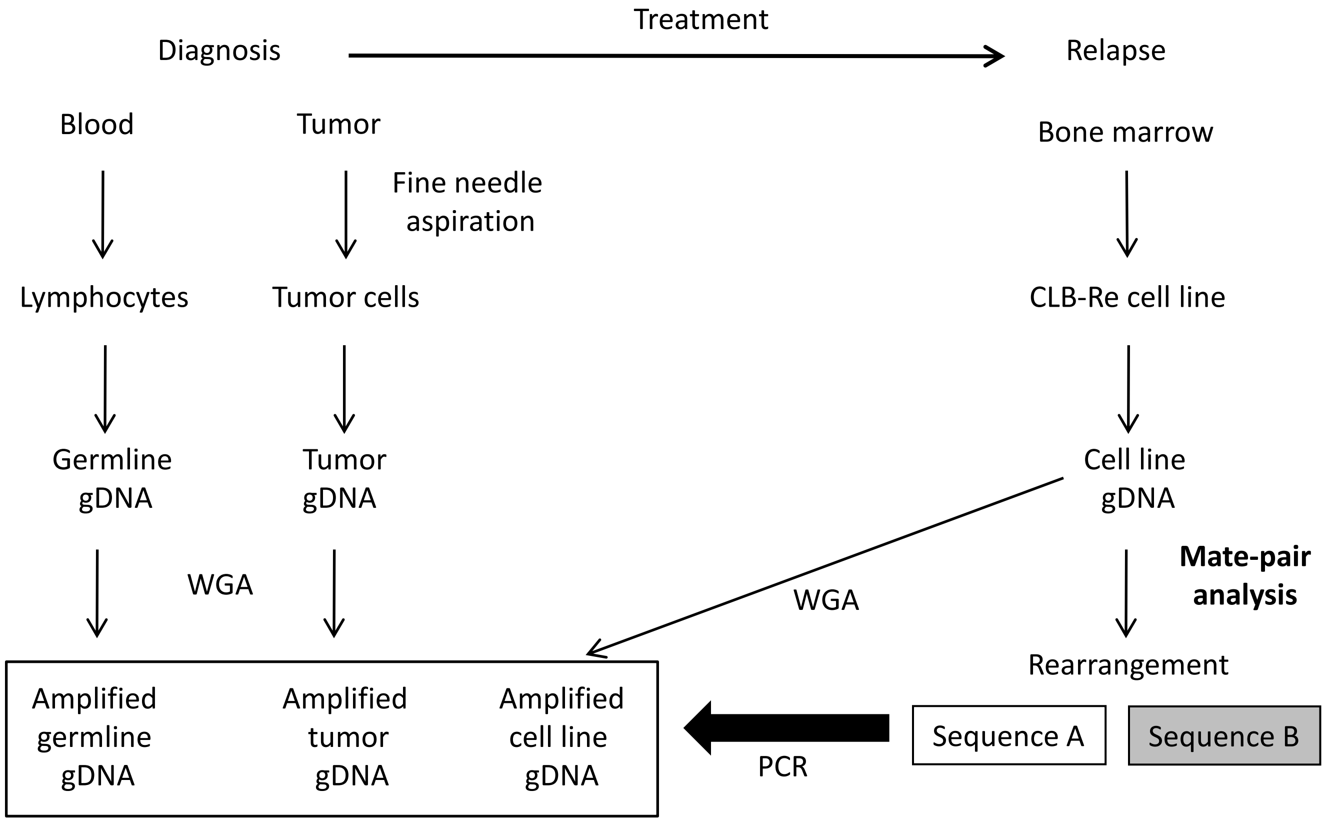


**B**

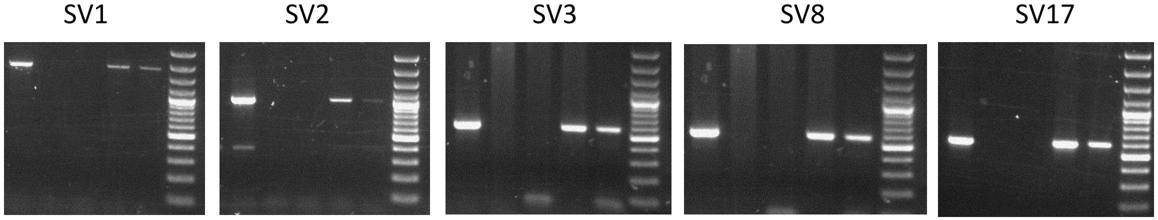


Order of samples from left to right: CLB-Re gDNA, $H_2O$, amplified-germline DNA, amplified-tumor DNA, amplified-cell line DNA
