# Supplementary material for: Breakpoint Features of Genomic Rearrangements in Neuroblastoma with Unbalanced Translocations and Chromothripsis
Source: PLoS One. 2013 Aug 26;8(8):e72182. doi: 10.1371/journal.pone.0072182 (PMC3753337; doi:10.1371/journal.pone.0072182)

Supplementary figure S3: Complex rearrangement between chromosomes 12 and 20 in CLB-Ga. The copy number profile for chromosome 20 together with a link between chromosomes 12 and 20 (SV7) suggested an unbalanced t(12,20) translocation. With a resolution of 30 kb no copy number change were observed in the corresponding region of chromosome 12. Analysis of intra-chromosomal SVs on chromosome 12 followed by Sanger sequencing revealed a complex structure of SV11. Altogether, our analysis predicts a rearrangement including a fragment from chromosome 20 (17.4 Mb, magenta) and three fragments from chromosome 12 (30 kb, 243 bp and 1.8 Mb, purple, turquois and blue, respectively).

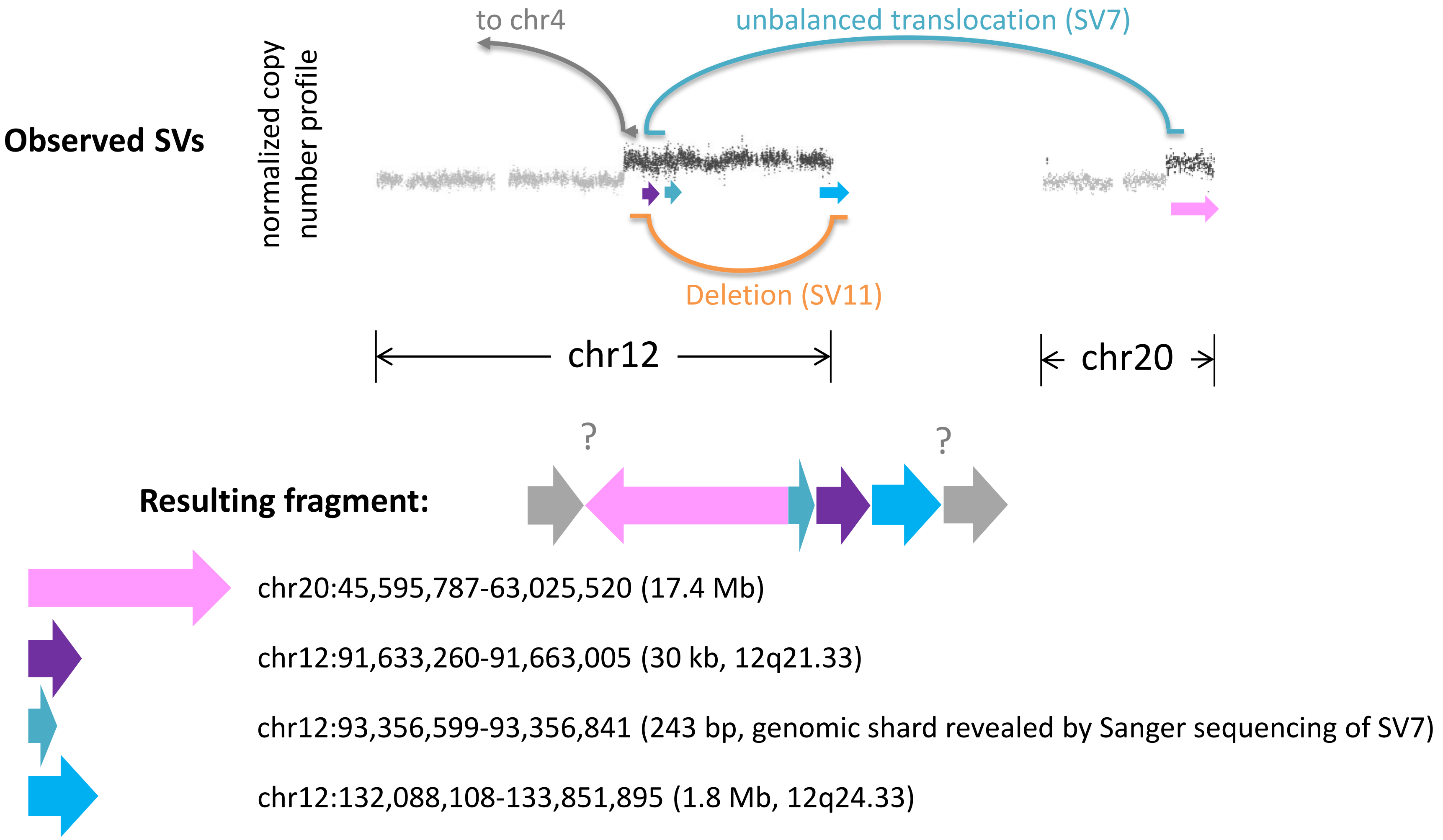

Supplement: Figure S3 — Complex rearrangement between chromosomes 12 and 20 in CLB-Ga. The copy number profile for chromosome 20 together with a link between chromosomes 12 and 20 (SV7) suggested an unbalanced t(12,20) translocation. With a resolution of 30 kb no copy number change were observed in the corresponding region of chromosome 12. Analysis of intra-chromosomal SVs on chromosome 12 followed by Sanger sequencing revealed a complex structure of SV11. Altogether, our analysis predicts a rearrangement including a fragment from chromosome 20 (17.4 Mb, magenta) and three fragments from chromosome 12 (30 kb, 243 bp and 1.8 Mb, purple, turquois and blue, respectively). (PDF) [file pone.0072182.s003.pdf]
