# Supplementary material for: Breakpoint Features of Genomic Rearrangements in Neuroblastoma with Unbalanced Translocations and Chromothripsis
Source: PLoS One. 2013 Aug 26;8(8):e72182. doi: 10.1371/journal.pone.0072182 (PMC3753337; doi:10.1371/journal.pone.0072182)

Supplementary figure S4: Predicted amplicons' structure in NB1141. A, normalized copy number profiles for chromosomes 1 and 2 determined by the FREEC method. B, Scheme of the potential structure of two amplicons involving regions located on chromosomes 1 and 2. The upper part represents the normal position of the regions involved in the amplicons, whereas the middle and lower parts deciphers the organization of those regions within the two amplicons. The direction of the arrows indicates the orientation of each fragment. Amplicon A6 contains the *MYCN* gene.

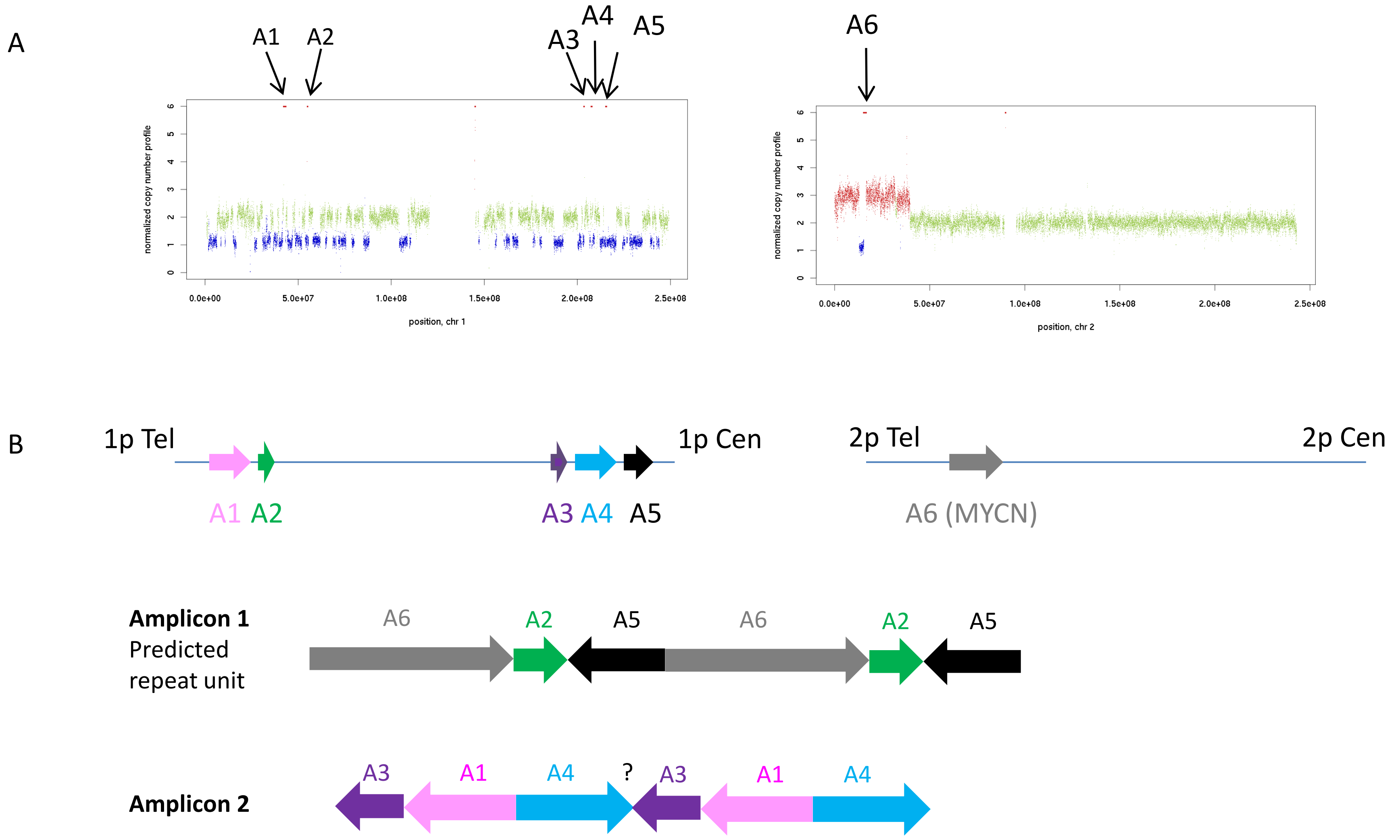

Supplement: Figure S4 — Predicted amplicons’ structure in NB1141. A, normalized copy number profiles for chromosomes 1 and 2 determined by the FREEC method. B, Scheme of the potential structure of two amplicons involving regions located on chromosomes 1 and 2. The upper part represents the normal position of the regions involved in the amplicons, whereas the middle and lower parts deciphers the organization of those regions within the two amplicons. The direction of the arrows indicates the orientation of each fragment. Amplicon A6 contains the MYCN gene. (PDF) [file pone.0072182.s004.pdf]
