# Supplementary material for: Breakpoint Features of Genomic Rearrangements in Neuroblastoma with Unbalanced Translocations and Chromothripsis
Source: PLoS One. 2013 Aug 26;8(8):e72182. doi: 10.1371/journal.pone.0072182 (PMC3753337; doi:10.1371/journal.pone.0072182)
Supplement: Figure S5 — Junctions identified at the base pair level in CLB-Ga, CLB-Re, NB1141 and NB1142. (PDF) [file pone.0072182.s005.pdf]

Supplementary figure S5: Junctions identified at the base pair level in CLB-Ga, CLB-Re, NB1141 and NB1142.

- **CLB-GA SV1 (17p13; 1p36)**

Chr A chr17:8162035-8162094, (+)

```
TGGCTCATGCCTGTAATCCCAACACTTTGGGAGGCTGAGGTGGGCAGATCACGAGGTCAG
||||||||||||||||||||||||||||||||||||||||
TGGCTCATGCCTGTAATCCCAACACTTTGGGAGGCTGAGGAGAGCTGATCCCGAGGTCAG
                                    ||||||||||||||||||||||||
TGGTTCACAGCTGTAATCCCAGCATTTTGGAAGGCGGAGGAGAGCTGATCCCGAGGTCAG
```

Chr B chr1:27516287-27516346, (+)

- **CLB-GA SV2 (Telomeric Seq ;2p14.2)**

Chr A Telomeric sequence (TTAGGG repetition)

```
TTAGGGTTAGGGTTAGGG.TTAGGGTTAGGGTTAGGGTTAGGGTTAGGG
|||||||||||||||||| |||
TTAGGGTTAGGGTTAGGGGTTACTCCCAAATCCCCGTAGGCCTGCCCTTCCT
                      ||||||||||||||||||||||||||||||
TCCTGCACTCACATCCCAGCATCTCCCAAATCCCCGTAGGCCTGCCCTTCCT
```

Chr B chr2: 29953041-29953092 (-)

- **CLB-GA SV3 (4q31.1;3p14.2)**

Chr A chr4: 139766548-139766588, (-)

```
CCTCAGCCACATTGCACTGCTTAAACGAGGTTAAATGCCTT
|||||||||||||||||||||
CCTCAGCCACATTGCACTGCTATGTCTATTAGAGAATCCCT
                     |||||||||||||||||||||
 TTTGTGAAATTGTAGAAAGCTATGTCTATTAGAGAATCCCT
```

Chr B chr3:62979907-62979947, (+)

- **CLB-GA SV4 (12q21.1;4p15.2)**

Chr A chr12: 72621910-72621950 (-)

```
TGAGGAAAAGAAATGTGGGGTTGGAGCCCCCACACAGAGTC
|||||||||||||||||||||
TGAGGAAAAGAAATGTGGGGTGAAGAAGGTCTGAGGTAGTT
                   ||||||||||||||||||||||
AGTAGTTTTTTCTAATTCTGTGAAGAAGGTCTGAGGTAGTT
```

Chr B chr4: 25288247-25288287, (+)

- **CLB-GA SV5 (4q34;17q21)**

Chr A chr 4: 175025131-175025171, (+)

```
GATAACAAAAACTTCTAAGGCTTTTCAAAGTAAGTCTTTCG
|||||||||||||||||||||
GATAACAAAAACTTCTAAGGCCACCTCTTTCACGACGGTTC
                    |||||||||||||||||||||
GCTCATACAATCTCATGCACCCACCTCTTTCACGACGGTTC
```

Chr B chr 17: 47031727-47031767, (+)

- **CLB-GA SV6 (11p13;5q33.1)**

Chr A chr 11: 41211773-41211813, (+)

```
AGCCTGAAGACAGACGGAGGACAAGAGAGAGGAAGGGTCAC
|||||||||||||||||
AGCCTGAAGACAGACGGGAGGAGGGTATCTGGCAGCTCCCA
               ||||||||||||||||||||||||||
CTCTGCACCTGGGATGGGAGGAGGGTATCTGGCAGCTCCCA
```

Chr B : chr 5: 149499738-149499778, (+)

- **CLB-GA SV7 – junction 1 (20q13.12;12q21.33)**

```
Chr A chr20: 45595767-45595807, (-)

                    AACAAAAATTAGCTGGATGTGGTGGTGTGTGCCTGTAGTCC
                    |||||||||||||||||||||
                    AACAAAAATTAGCTGGATGTGAGCCACCATGCCTATTTTTT
                                   |||||||||||||||||||||||||
                    ATTGCTGGGATTCCAGATGTGAGCCACCATGCCTATTTTTT

Chr B chr12: 93356584-93356624, (+)
```

- **CLB-GA SV7 – junction 2 (12q21.33;12q21.33)**

```
Chr A chr 12: 93356821-93356861, (+)

                    AGGTCAGCATATTTCTTTGAATTTAGATATGTGGAAATACT
                    |||||||||||||||||||||
                    AGGTCAGCATATTTCTTTGAACGAAAATATGTCAGTAGTTG
                                       ||||||||||||||||||||||
                    TGAGGCTTTAAACTGGATAAACGAAAATATGTCAGTAGTTG

Chr B chr12: 91633241-91633281, (+)
```

- **CLB-GA SV8 (5q33.1; 5q33.1)**

```
Chr A chr 5: 149549537-149549576, (+)

                    ATATGTAGGATAAACCCCACATTCCTTCCTGAGGTCTACAA
                    |||||||||||||||||||||
                    ATATGTAGGATAAACCCCACAAAGTAGCAGGAGAATGGCTT
                                       ||||||||||||||||||||||
                    TTAACTTGCCTTATCTAGGCAAAGTAGCAGGAGAATGGCTT

Chr B chr 5: 149551285-149551325, (-)
```

- **CLB-GA SV9 (6q13; 6q16.2)**

Chr A chr 6: 75177056-75177096, (+)

```
CTAGTTGTGTCTATTTGGCCATGTTGACTCCTCCTTCTGGG
|||||||||||||||||||||
CTAGTTGTGTCTATTTGGCCAGAGCCTCAGTTTTGACATTC
                 ||||||||||||||||||||||||
AAAGCTCTAGCTCCGAAGCCAGAGCCTCAGTTTTGACATTC
```

Chr B chr 6: 99278172-99278212, (+)

- **CLB-GA SV10 (11q14.1; 11q24.3)**

Chr A chr 11: 83456896-83456936, (+)

```
AGAATCTACAAAGAACTCAAACAAATTTAGAAGAAAAAAAC
|||||||||||||||||||||
AGAATCTACAAAGAACTCAAAGCATCTTTGCCGGAGCAAAT
                     ||||||||||||||||||||
ACGGCTGTGTTTCTGGATCTGGCATCTTTGCCGGAGCAAAT
```

Chr B chr 11: 129223440-129223480, (+)

- **CLB-GA SV11 (12q21.33; 12q24.33)**

Chr A chr 12: 91662985-91663025, (+)

```
GAAAAAGGTATATTTTTTACTAAGTCTGGTAGGATGAATAG
|||||||||||||||||||||
GAAAAAGGTATATTTTTTACTTGTTCTCATCTGAAGGCTCA
                   ||||||||||||||||||||||
AACCAAGGTGCTGTCTGGCCTTGTTCTCATCTGAAGGCTCA
```

Chr B chr 12: 132088089-132088129, (+)

- **CLB-RE SV1 - junction 1 (1q41;7q31.33)**

Chr A chr 1: 223989291-223989348 (+)

```
GAATGCAAAGATCACTAACACAAGGAGCCTCTGCTATTTTCACTTTTAATCTCCCAAT
|||||||||||||||||||||
GAATGCAAAGATCACTAACACTAAGATCACTAACAAAGATCATTATAATAAGGACTTT
                                     |||||||||||||||||||||
  AGTTTATAGGAGGCCAGATGATATAGGGTCTTGTTGATCATTATAATAAGGACTTT
```

Chr B chr7: 126004890-126004945 (+)

- **CLB-RE SV1 – junction 2 (7q31.33;7q31.33)**

Chr A chr 7: 126004948-126004988 (+)

```
ATCTGTGCTGTACAATACTGTGGCCCTCGGCCAAATAGAGC
|||||||||||||||||||||
ATCTGTGCTGTACAATACTGTGAAATTAGTATTATGAGAAA
                     ||||||||||||||||||||
AACCCTGACTAATACAAGGAGGAAATTAGTATTATGAGAAA
```

Chr B chr 7: 126004604-126004644 (+)

- **CLB-RE SV2 (2p21;3p12.3)**

Chr A chr 2: 43811166-43811206 (+)

```
TTGTCTCAATTAAAAAAAAAAAAAAAAAAAAAAGGAAAAAT
|||||||||||||||||||||
TTGTCTCAATTAAAAAAAAAAGGATGTGGAGAGATGGGAAT
                 ||||||||||||||||||||||||
GGATCTTATCAATTTTGAAAAGGATGTGGAGAGATGGGAAT
```

Chr B chr 3: 76179229-76179269 (+)

- **CLB-RE SV3 (2p21;3p14.2)**

Chr A chr 2: 43966801-43966841 (+)

```
TCTGCATCAAGAACACTTTGTCATCTGGCTCCACTGGCCCG
|||||||||||||||||||||
TCTGCATCAAGAACACTTTGTGGATGTGGAGAGATGGGAAT
                   ||||||||||||||||||||||
CAATGCAGATGGGTGTAAAGTGCAACCCCCCAAATCTCCAA
```

Chr B chr 3: 58586942-58586982 (+)

- **CLB-RE SV4 (3p12.3;2p22.3)**

Chr A chr 3: 77111808-77111851 (+)

```
TATCTGGGCATCCAGTGGCCCATAAATTGGAGTTCCACCACACT
|||||||||||||||||||||
TATCTGGGCATCCAGTGGCCCCCCATCCTTGTGCCAGATAATCC
                   |||||||||||||||||||||||||
CCTCTTTTACTTATAAGGACCCCCATCCTTGTGCCAGATAATCC
```

Chr B chr 2: 33828528-33828571 (+)

- **CLB-RE SV5 (2p23.3;3p12.3)**

Chr A chr 2: 26670783-26670823 (+)

```
GTCAGTTCTTGCTGTTAATATTTCTCTGGACCAGGCGCCGT
|||||||||||||||||||||
GTCAGTTCTTGCTGTTAATATATGCTCACTGGATCCCATTC
                     ||||||||||||||||||||
TATCTGTTTTTCTCACAAGAAATGCTCACTGGATCCCATTC
```

Chr B chr 3: 76235665-76235705 (+)

- **CLB-RE SV6 (2p23.3;3p14.2)**

Chr A chr 2 : 26330831-26330871 (+)

```
ATTACTATGTGCCCTCTTAGAACTACAGAAATAAAAAAGTG
|||||||||||||||||||||
ATTACTATGTGCCCTCTTAGAGCTTTCTAAACCATCAAGGG
                  |||||||||||||||||||||||
TGAACTACTACATGAGAAAGAGCTTTCTAAACCATCAAGGG
```

Chr B chr 3 : 58729858-58729898 (-)

- **CLB-RE SV7 (2p24.1;3p12.3)**

Chr A chr 2 : 23741028-23741068(+)

```
CTTCCTCAGCCCCTCCGTGGGGACCCAGCCCGGAGGGAACC
|||||||||||||||||||||
CTTCCTCAGCCCCTCCGTGGGACTCAGCAAATAAGAACACG
                     ||||||||||||||||||||
TATTGACTTGTTCATAAATTCACTCAGCAAATAAGAACACG
```

Chr B chr 3 : 77218310-77218350 (-)

- **CLB-RE SV8 – junction 1 (19p13.12;4q13.1)**

Chr A chr 19: 14948898-14948952 (+)

```
TTATTGTGAAAAGTAAGCAATATTTTAATGCTTGGAAAGTTACTGGAACAGAATA
|||||||||||||||||||||
TTATTGTGAAAAGTAAGCAATTATCAATTGTATCTGAAGACCCAAAGCATCTAAT
                              |||||||||||||||||||||||||
AGTGAGACAGATAGTATGAATTGATAATAATATCTGAAGACCCAAAGCATCTAAT
```

Chr B chr4: 60546141-60546195 (+)

- **CLB-RE SV8 – junction 2 (4q13.1; 4q13.1)**

Chr A chr 4 : 60546210-60546250 (+)

```
ATAGCATACTAGCTAAATTTCTGCTCACAATAAAGCTAATA
|||||||||||||||||||||
ATAGCATACTAGCTAAATTTCAGATAAGCTGAAACTTTAAA
                    |||||||||||||||||||||
ACGGTAATTTTACTGATGCCCAGATAAGCTGAAACTTTAAA
```

Chr B chr 4 : 60575220-60575260 (+)

- **CLB-RE SV9 (11q14.1; 14q32.33)**

Chr A chr 11 : 78544356-78544396 (+)

```
CAGCAATTTTTGATTGTCATGTGATGTTCTCTACTACTTCC
|||||||||||||||||||||
CAGCAATTTTTGATTGTCATGCCACCAGGATGAATAACTTC
                    |||||||||||||||||||||
TCCATTTTGTCGGTGGGCTGGCCACCAGGATGAATAACTTC
```

Chr B chr 14 : 104177420-104177460 (+)

- **CLB-RE SV10 (19p13.3;17q12)**

Chr A chr19:4789803-4789843 (-)

```
TGGCAGGCGCCTGTAGTCCCAGCTACTCGGGAGGCTGAGGC
||||||||||||||||||||
TGGCAGGCGCCTGTAGTCCCGCTTCCCCCTCTAACCCAGGA
                 ||||||||||||||||||||||||
GACTTCCCCCCTCCCCACCCGCTTCCCCCTCTAACCCAGGA
```

Chr B chr 17 : 37803114-37803154 (+)

- **CLB-RE SV11 (2p25.3; 3p14.2)**

Chr A chr 2: 488988-489028(+)

```
TCTCCCTGCTCCGGGCACCCTCCTCACTGAGGCAAAACATA
|||||||||||||||||||||
TCTCCCTGCTCCGGGCACCCTAGTGTGGCTCATTTTCTTCC
                    |||||||||||||||||||||
GCTCTCAGCGTTTGAAGGGATAGTGTGGCTCATTTTCTTCC
```

Chr B : chr 3: 59822039-59822079 (-)

- **CLB-RE SV12 (2p25.3; 3p14.2)**

Chr A chr2: 2200049-2200089 (+)

```
GGTAAAGGATTTTAGATACATTAAAAATAGAAGCACTTGTA
|||||||||||||||||||||
GGTAAAGGATTTTAGATACATCCAGCTTGCAATGCAGACCT
                    |||||||||||||||||||||
TAGTGATAAGCAAGGAACATTCCAGCTTGCAATGCAGACCT
```

Chr B chr3: 60006341-60006381 (+)

- **CLB-RE SV13 (3p14.2;2p25.1)**

Chr A chr 3: 60255757-60255797 (-)

```
AATCTGATTGGCTGGCCCAGTGTAGAGTCAGATACACCCCC
|||||||||||||||||||||
AATCTGATTGGCTGGCCCAGTAGAACTGTGGTGTTCAAAGG
                 ||||||||||||||||||||||||
TCAAAGGCTATGGTGAGCAGTAGAACTGTGGTGTTCAAAGG
```

Chr B chr 2: 11212326-11212366 (+)

- **CLB-RE SV14 (3p14.2;2p24.3)**

Chr A chr 3: 60003286-60003326 (+)

```
AATTACTGAGAATTATGGACCTTCTCACAACACATGATTCA
|||||||||||||||||||||
AATTACTGAGAATTATGGACCCAGCGCAGACCTGGAGGCGC
                    |||||||||||||||||||||
AGTGGGAGCAGGCGCCCCGGCCAGCGCAGACCTGGAGGCGC
```

Chr B chr 2: 14772867-14772907 (+)

- **CLB-RE SV15 – junction 1 (2p24.3; 2p24.3)**

Chr A chr2: 15191853-15191905 (+)

```
ATTTTATCACATATCATTTGATGCCATTCTGTCACCGTGGGACTTCCATCAAG
|||||||||||||||||||||
ATTTTATCACATATCATTTGACACCTGGATATATTATTTATGTCCACAATATA
                           ||||||||||||||||||||||||||
TATGTGTTTCTTCAGTAATAACAGAAAGATATATTATTTATGTCCACAATATA
```

Chr B chr2: 24207372-24207424 (-)

- **CLB-RE SV15 – junction 2 (2p24.3; 3p14.2)**

Chr A chr 2: 24207267-24207307 (-)

```
TACTGGTATTATTTGAGTAGTGCTTTACAGAATGGATAATT
|||||||||||||||||||||
TACTGGTATTATTTGAGTAGTCCTTCACCTCCACAACCAAC
                    |||||||||||||||||||||
GTGGAAATGTACCCAGCCACTCCTTCACCTCCACAACCAAC
```

Chr B chr3: 59851487-59851527 (+)

- **CLB-RE SV16 (2p22.3; 3p14.2)**

Chr A chr 2: 35051627-35051667 (+)

```
CAGTTCATGCTGCTACTACCAGAGTATGTTATTTATGCCAC
|||||||||||||||||||||
CAGTTCATGCTGCTACTACCAATTAATTCTGTGTTGTATTA
                     ||||||||||||||||||||
TTTTATTAGTTCAGAAGAATGATTAATTCTGTGTTGTATTA
```

Chr B chr 3: 60006259-60006299 (-)

- **CLB-RE SV17 (4q34.2; 4q35.1)**

Chr A chr 4 : 177386850-177386890 (+)

```
CAGTAACTATCATTCTCACCTGGATTCCTGCAACTATTTTT
|||||||||||||||||||||
CAGTAACTATCATTCTCACCTAAGACGTTTTATTTAATAAC
                     ||||||||||||||||||||
GTTAGGTGGTTGAGGGCATGGAAGACGTTTTATTTAATAAC
```

Chr B chr 4 : 184907641-184907681 (+)

- **CLB-RE SV18 (3p14.1;2p23.2)**

Chr A chr3: 65182902-65182942 (-)

```
TGGCCAATTTAATGTCAGGATTTCTCAAGGGGCTTTAAAAT
||||||||||||||||||||
TGGCCAATTTAATGTCAGGAGAATTCCTGGGTCCATCTGTC
                     ||||||||||||||||||||
TCACTGGCTGAACCAGGAAGCAATTCCTGGGTCCATCTGTC
```

Chr B chr2: 29569636-29569676(+)

- **CLB-RE SV19 (2p23.2; 3p12.3)**

Chr A chr2: 29704228-29704268(-)

```
GAATCTTATGATAAAGACTGAAAAATAATTGTCTTCCCTTC
|||||||||||||||||||||
GAATCTTATGATAAAGACTGATTGAATGAGCATCTACTACA
                    |||||||||||||||||||||
ATTTCATTTATTGAACAAATATTGAATGAGCATCTACTACA
```

Chr B chr3: 74522703-74522742(+)

- **CLB-RE SV20 (3p14.2;2p23.2)**

Chr A chr3: 61205732-61205772 (+)

```
TTAAGTCTTTAACAATTATTTACATACCTAGGAAAGAGTCT
||||||||||||||||||||
TTAAGTCTTTAACAATTATTACAGAGCATGAGGTTAATCAG
                    |||||||||||||||||||||
AATCTAAAATAAGCAAGAACACAGAGCATGAGGTTAATCAG
```

Chr B chr2: 29492536-29492576(+)

- **CLB-RE SV21 (2p23.2; 2p22.3)**

Chr A chr2: 29490953-29490994(+)

```
TGCTTAGAAGGGCAACAGTATCGACTTTATGTGGAAGCATTT
||||||||||||||||||||
TGCTTAGAAGGGCAACAGTAAGCAAAGTCAGAGCAAGCATCT
                     |||||||||||||||||||||
AGCTTTTACTCGTGGCAGAAGGCAAAGTCAGAGCAAGCATCT
```

Chr B chr2: 33302520-33302561 (+)

- **CLB-RE SV22 (2p23.2; 2p21)**

```
Chr A chr2: chr2:29750377-29750419(+)

                         TTAGTTTGTTTTTTTGAGACAGGGTCTTGCTCTGTCACCCAGG
                         |||||||||||||||||||||||
                         TTAGTTTGTTTTTTTGAGACAGGCATCCCAGGGTAGTGCCAGC
                                              ||||||||||||||||||||||
                         CATATACAGCCTAAATTCCCCGGCATCCCAGGGTAGTGCCAGC

Chr B chr2 : 42156046-42156088 (+)
```

- **CLB-RE SV23 (2p23.2; 2p22.2)**

```
Chr A chr2: 29793205-29793246(+)

                          TGTAGTCATCTGTGCCTATGAAGTTGGCTTAATTTTTAGGGC
                          ||||||||||||||||||||||
                          TGTAGTCATCTGTGCCTATGAAAAATCCAATGAGGCCAGGCA
                                              ||||||||||||||||||||||
                          CAGCCTGTTTTTGCTTTTTTAAAAATCCAATGAGGCCAGGCA

Chr B chr2: 37040278-37040319 (+)
```

- **NB1141 SV1 (1q32.1; 1q32.1)**

```
Chr A Chr1: 205496895 – 205496939 (+)

            CCCACATTTCTCTTCCCCCTCCCCCAGATTTTCATGTTCCAGCTG
            |||||||||||||||||||||||||
            CCCACATTTCTCTTCCCCCTCCCCCGACCTCCTGTGCAGGGCCGT
                                 ||||||||||||||||||||||||
            CATCACCTACCGCTTCCAGGGCCCCGACCTCCTGTGCAGGGCCGT

Chr B Chr1: 206224725 – 206224769 (+)
```

- **NB1141 SV2 (1p36.11; 1p33)**

```
Chr A chr1: 26494130-26494173 (+)

            TGAGATGGAGTCTTGCTCTGTCGCCCAGGCTGGAGTGCAATGGC
            ||||||||||||||||||||
            TGAGATGGAGTCTTGCTCTGGGTGGGTGGTGGGCAGGGCCTGTA
                                ||||||||||||||||||||||||
            AGGGAGAGAGGTCATAGTGAGGTGGGTGGTGGGCAGGGCCTGTA

Chr B Chr1: 48276716 – 48,276,759 (+)
```

- **NB1141 SV3 - junction 1 (1p33; 1p33)**

```
Chr A Chr1: 46872108–46872148 (+)

            CAGGCCTTGGAGCCCCTGTCTCCTGAGAACCGTCCCCCAGG
            ||||||||||||||||||||
            CAGGCCTTGGAGCCCCTGTCGCCTGAGGATTTCCAAATGCA
                                |||||||||||||||||||||
            GAAAAGGAAAAAAAAAAAAAGCCTGAGGATTTCCAAATGCA

Chr B Chr1: 46872207–46872247 (–)
```

- **NB1141 SV3 - junction 2 (1p33; 1p13.3)**

Chr A Chr1: 46872114-46872157 (-)

```
CCTCAGAGGCCTGGGGGACGGTTCTCAGGAGACAGGGGCTCCAA
||||||||||||||||||||||||
CCTCAGAGGCCTGGGGGACGGTTCAGAGGCTTCCTCGGAGTTGC
                     |||||||||||||||||||||||
ACGCAGGAGGCCACAGGGGGCTTCAGAGGCTTCCTCGGAGTTGC
```

Chr B Chr1: 110731510-110731553 (-)

- **NB1141 SV3 - junction 3 (1p13.3; 1p13.3)**

Chr A Chr1: 110731389-110731438 (-)

```
AAGCAGGTCCTCGGAGAAGCAAGCCTCGGATCAGGAGAAGGGCAGTATCC
|||||||||||||||||||||||||||
AAGCAGGTCCTCGGAGAAGCAAGCCTCTGAAGCCCCCTGTGGCCTCCTGC
                    ||||||||||||||||||||||||||||||
GCCAGGGAGCAACTCCGAGGAAGCCTCTGAAGCCCCCTGTGGCCTCCTGC
```

Chr B Chr1: 110731502-110731551 (+)

- **NB1141 SV4 - (1q25.2; 1q44)**

Chr A Chr1: 176024185-176024232 (+)

```
CTCTTCCACAATAGAGTGATTTTTCTCCTACCTTACTGACAACTCCAT
||||||||||||||||||||||||||||
CTCTTCCACAATAGAGTGATTTTTCTCCACTATTGTATATGTTTTAAG
                    ||||||||||||||||||||||||||||
TTCCACAAACCTATCTTGAATTTTCTCCACTATTGTATATGTTTTAAG
```

Chr B Chr1: 244019614-244019661 (+)

- **NB1141 SV5 - (1p22.1; 1p31.3)**

Chr A Chr1: 94368759-94368798 (-)

```
AAATCAGCAATTATGCTCCTTAGTATCTACCCAAAGAAGC
||||||||||||||||||||
AAATCAGCAATTATGCTCCTCCTTGGAATTCCTTTGCCCC
                    ||||||||||||||||||||
TCACAAAAGAAGCTGCTTTCCCTTGGAATTCCTTTGCCCC
```

Chr B Chr1: 61858529-61858568 (+)

- **NB1141 SV6 - (1q25.3; 1q44)**

Chr A Chr1: 183410541-183410583 (+)

```
TCACTTGATAACGCAAGAGAATGATAGTGCTGGGAAATCTCTC
|||||||||||| |||||||
TCACTTGATAACACAAGAGAGATTGAGTATGTTCTCTCTGCAT
                       ||||||||||||||||||||
GCTTGGTGCCATCCCCAGGATAATGAGTATGTTCTCTCTGCAT
```

Chr B Chr1: 246885624-246885666 (-)

- **NB1141 SV7 - (1p13.2;1p33)**

Chr A Chr1: 113423711-113423754 (-)

```
CTCTTGTGGCCAGAGCGGCCCTGCAGCCTCTTGGCAGGATCCCG
||||||||||||||||||||||||
CTCTTGTGGCCAGAGCGGCCCTGCGGAAACCCAAAACCTCCGCA
                      ||||||||||||||||||||||
CCAGGGGCTAGGGGGAGGCCGGGCGGAAACCCAAAACCTCCGCA
```

Chr B Chr1: 48059054-48059097 (+)

- **NB1141 SV8 - (1p31.3; 1q42.12)**

Chr A Chr1: 64638431-64638471 (+)

```
TTGAACTGCCAAAACTTTATGACTTCATTTTCTTTCATAGT
|||||||||||||||||||||
TTGAACTGCCAAAACTTTATGGCGGAGGTTGCAGTGAGCCG
                    |||||||||||||||||||||
GAGAATTGGTTGTACTGGGAGGCGGAGGTTGCAGTGAGCCG
```

Chr B Chr1: 226586843-226586883 (-)

- **NB1141 SV9 - (1q31.1; 1q31.1)**

Chr A Chr1: 186321879-186321919 (+)

```
AAGAGCATTATAAGACATGGGTTCAAGTCCTAACCAACCCA
|||||||||||||||||||||
AAGAGCATTATAAGACATGGGGCTCCTGCTTCATTTCTTTC
                    |||||||||||||||||||||
GTGGCCCTACAGTACCATGTGGCTCCTGCTTCATTTCTTTC
```

Chr B Chr1: 186232785-186232825 (+)

- **NB1141 SV10 - (1p32.2;1p33)**

Chr A Chr1: 57895194-57895233 (+)

```
TGTTGCCTATGCTTTTGGTGCCATATCCAAAAAATCATTG
||||||||||||||||||||
TGTTGCCTATGCTTTTGGTGTCTAGTCCCAGCTCTACCAC
                    ||||||||||||||||||||
CAGAGGCAGGAGTCCTGGGTTCTAGTCCCAGCTCTACCAC
```

Chr B Chr1: 47079967-47080006 (+)

- **NB1141 SV11 - (1q31.3; 1q25.2)**

Chr A Chr1: 197116677-197116718 (+)

```
GCTGGTCTGGAACTCCTGGCCTCAAGTGATCCGCCTGCCTCC
|||||||||||||||||||||
GCTGGTCTGGAACTCCTGGCCACCTTCTCAGCCTCCTGTGCC
                    ||||||||||||||||||||||
ACTTACGTAGGTGCTTCTGACACCTTCTCAGCCTCCTGTGCC
```

Chr B Chr1: 176661912-176661953 (+)

- **NB1141 SV12 - (1q25.1;1p31.1)**

Chr A Chr1: 174930679-174930728 (+)

```
GGCTTAAAAACACAGCTAATTGGCCGGGCATGGTGGCTCTTGCCTGTAAT
|||||||||||||||||||||||||||||||||||||||
GGCTTAAAAACACAGCTAATTGGCCGGGCATGGTGGCTCACGCCTGTAAT
                    |||||||||||||||||||| |||||||||
ATAAATAAAAAGAAAGCTGGTGGCCGGGCATGGTGGCTCATGCCTGTAAT
```

Chr B Chr1: 78381012-78381061 (+)

- **NB1142 SV1 – junction 1 (19p13.42;6q22.31)**

Chr A chr 19:55654865-55654911 (-)

```
ACTTGGCCCCCTGGCCCAGAGACCCTTGGGGATTGTCCTCACCTCTT
|||||||||||||||||||||||||||
ACTTGGCCCCCTGGCCCAGAGACCCTTCTCAGCATCTCTGCCTCCAT
ACTTGGCCCCCTGGCCCAGAGACCGTTCTCAGCATCTCTGCCTCCAT
                    |||||||||||||||||||||||||||
AGCCGTACCCCTTCCAATCTGACCGTTCTCAGCATCTCTGCCTCCAT
```

Chr B chr6: 125180197-125180243 (+)

- **NB1142 SV1 – junction 2 (6q22.31;6q22.31)**

Chr A chr 6: 125180301-125180364 (+)

```
ACTAGCTTTCCTGCTTCTACTCTTGGCCCCATGTATTATAATAATAGATATATAGTAGATAATT
||||||||||||||||||||||||||||||||||||||||||||||
ACTAGCTTTCCTGCTTCTACTCTTGGCCCCATGTATTATAATAATATATATAATAGTTCTTCAC
ACTAGCTTTCCTGCTTCTACTCTTGGCCCCATCTATTATAATAATATATATAATAGTTCTTCAC
                  ||||||||||||||||||||||||||||||||||||||||||||||
ATTATATTTCCGGCTTCCACTCTTGGCCCCATCTATTATAATAATATATATAATAGTTCTTCAC
```

Chr B chr6: 125180864-125180927 (+)

- **NB1142 SV2 (6q23.3;19q13.42)**

Chr A Chr6: 136611724-136611764 (-)

```
CAAGCTACGTTGGGCTGTGTTCCTCGGCTGCCACAAATCAT
|||||||||||||||||||||
CAAGCTACGTTGGGCTGTGTTTGGCTGCCTCCATCTGCAGT
                    |||||||||||||||||||||
CCTCCTCTAGCCCTTTCCCCTTGGCTGCCTCCATCTGCAGT
```

Chr B Chr19: 55556782-55556822 (+)

- **NB1142 SV3 (19q13.42;6p21.2)**

Chr A Chr19: 55577347-55577389 (+)

```
AAGTGCTGGGATTACAAAGCGTGAGCCACTGCGCCCAGCTACC
|||||||||||||||||||||||
AAGTGCTGGGATTACAAAGCGTGGCTTTACCTCTTCTTTTTTC
                    |||||||||||||||||||||||
GGGGCTAGCCTCTTACAGCAGTGGCTTTACCTCTTCTTTTTTC
```

Chr B Chr6: 37524821-37524863 (+)

- **NB1142 SV4 (6p21.2;19q13.42)**

Chr A Chr6: 160534011-160534053 (+)

```
TAAACTACACAGTCCATGGTACTTTATGGCAGCCTAAGCAAAC
|||||||||||||||||||||||
TAAACTACACAGTCCATGGTACTGGTTTTGAACTCCTGACCTC
                     ||||||||||||||||||||||
GGTTTCACCATGATGCCCAAGCTGGTTTTGAACTCCTGACCTC
```

Chr B Chr19: 55654316-55654358 (-)

- **NB1142 SV5 – junction 1 (6p21.31;19q13.42)**

Chr A Chr6: 35145886-35145939 (+)

```
GCAGGTTTAAAGGACCCAGGAAGGTACCCTCCTGGGATAGCCCTTATGACCTGA
|||||||||||||||||||||
GCAGGTTTAAAGGACCCAGGAGGTCCCAGTCTAGTAGGGGAATAGGAAAGGTAG
                               |||||||||||||||||||||||
ACCACTTGCCCGGGGCCCCACCCCTCATTCTTAGTAGGGGAATAGGAAAGGTAG
```

Chr B Chr19: 55600503-55600556 (+)

- **NB1142 SV5 – junction 2 (19q13.42;19q13.42)**

```
Chr A Chr19: 55600539-55600586 (+)

                GGGGAATAGGAAAGGTAGGCAATTCCCCCAAACCTCTTTGCTCCATTC
                ||||||||||||||||||||||||||||
                GGGGAATAGGAAAGGTAGGCAATTCCCCTACTAAGAATGAGGGGTGGG
                                     ||||||||||||||||||||||||||
                GGGGAATTGCCTACCTTTCCTATTCCCCTACTAAGAATGAGGGGTGGG

Chr B Chr19: 55600519-55600566 (-)
```
