# Supplementary material for: Breakpoint Features of Genomic Rearrangements in Neuroblastoma with Unbalanced Translocations and Chromothripsis
Source: PLoS One. 2013 Aug 26;8(8):e72182. doi: 10.1371/journal.pone.0072182 (PMC3753337; doi:10.1371/journal.pone.0072182)

Supplementary figure S6: Copy number profiles for the primary tumor NB1141 (**A**) and xenografted tumor (**B**) obtained using Roche NimbleGene CGH microarrays. Chromosome 1 of the NB1141 tumor has the signature of chromothripsis (Figure 3A-C). Here, we demonstrate that the xenografted tumor profile on chromosome 1 is identical to the one of the primary tumor with an exception for the amplicon structure A3-A1-A4 (see details in Supplementary figure 4) which is not present in the xenografted tumor. Thus, the xenographed sample can be used for validation of expression of abnormal RNA transcripts resulting from the chromothripsis with RNA-seq.

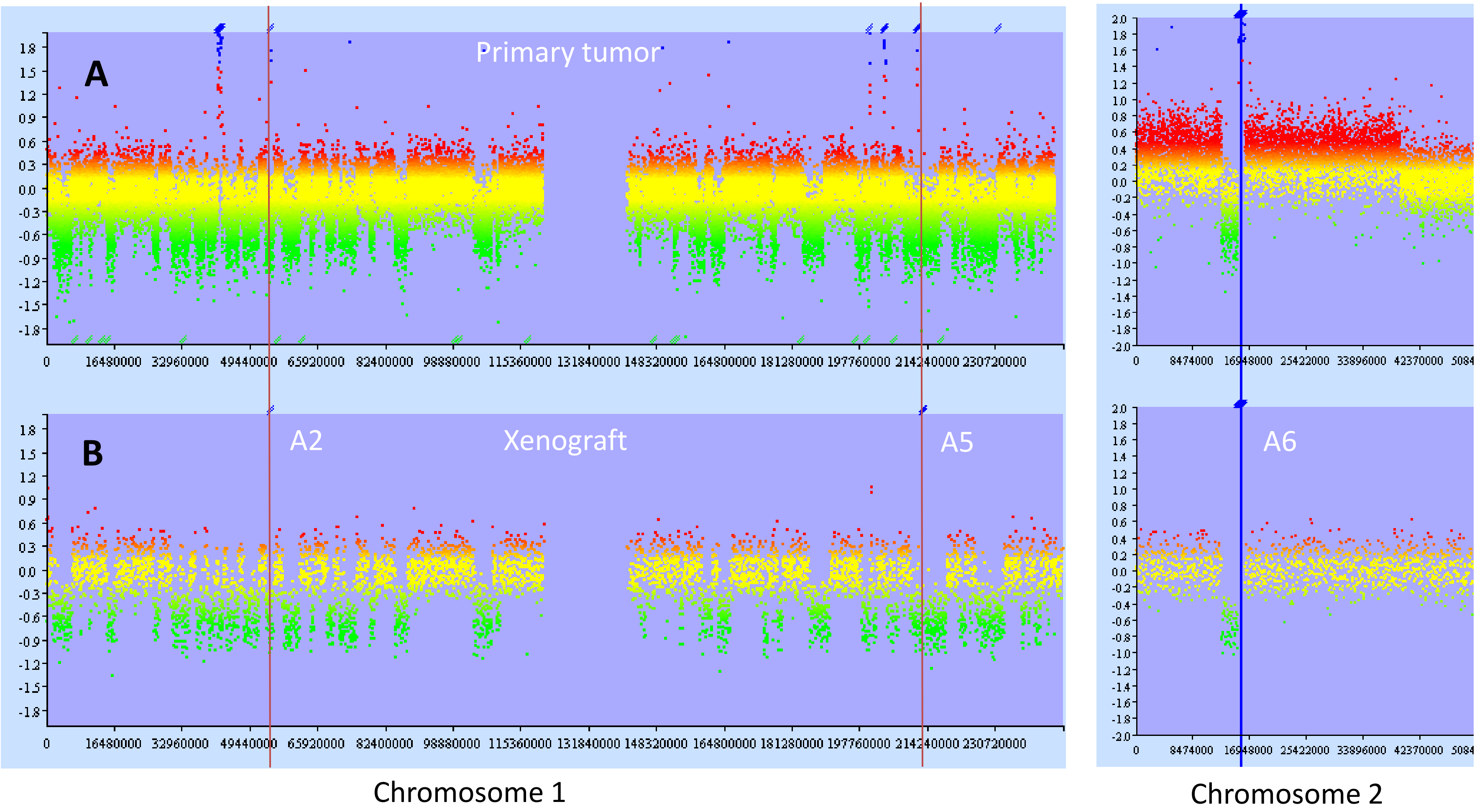

Supplement: Figure S6 — Copy number profiles for the primary tumor NB1141 (A) and xenografted tumor (B) obtained using Roche NimbleGene CGH microarrays. Chromosome 1 of the NB1141 tumor has the signature of chromothripsis (Figure 3A–C). Here, we demonstrate that the xenografted tumor profile on chromosome 1 is identical to the one of the primary tumor with an exception for the amplicon structure A3-A1-A4 (see details in figure S4) which is not present in the xenografted tumor. Thus, the xenographed sample can be used for validation of expression of abnormal RNA transcripts resulting from the chromothripsis with RNA-seq. (PDF) [file pone.0072182.s006.pdf]
