# Supplementary figures and images for: Breakpoint Features of Genomic Rearrangements in Neuroblastoma with Unbalanced Translocations and Chromothripsis
Source: PLoS One. 2013 Aug 26;8(8):e72182. doi: 10.1371/journal.pone.0072182 (PMC3753337; doi:10.1371/journal.pone.0072182)

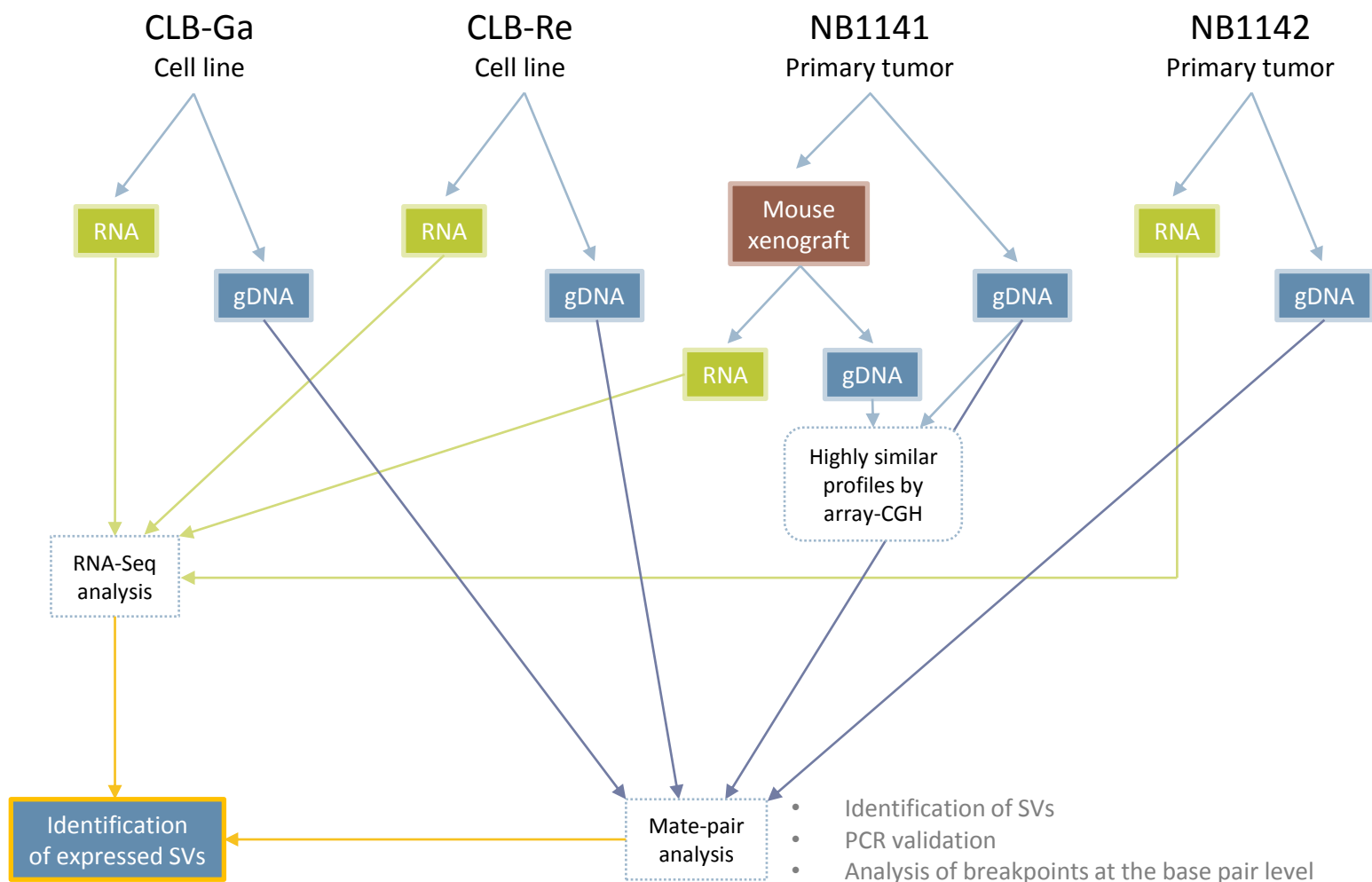

**Supplementary Figure S7.** Data processing workflow for the four analyzed NB samples.

Supplement: Figure S7 — Data processing workflow for the four analyzed NB samples. (PDF) [file pone.0072182.s007.pdf]

**Supplementary Figure S10.** Observed distribution of the insert sizes.

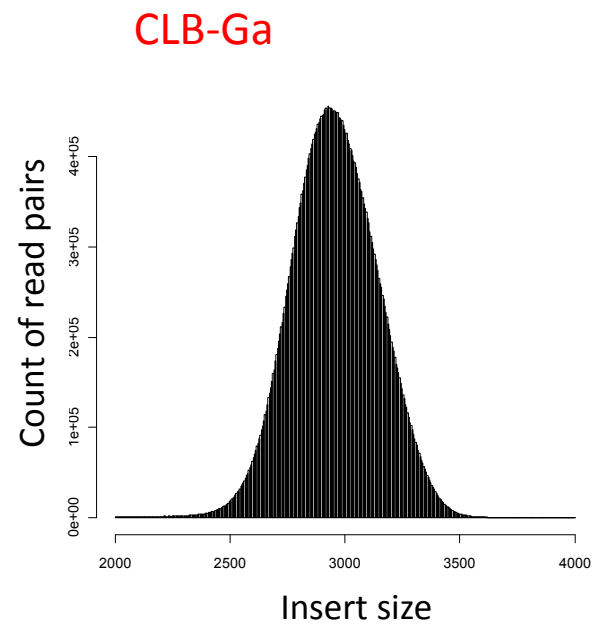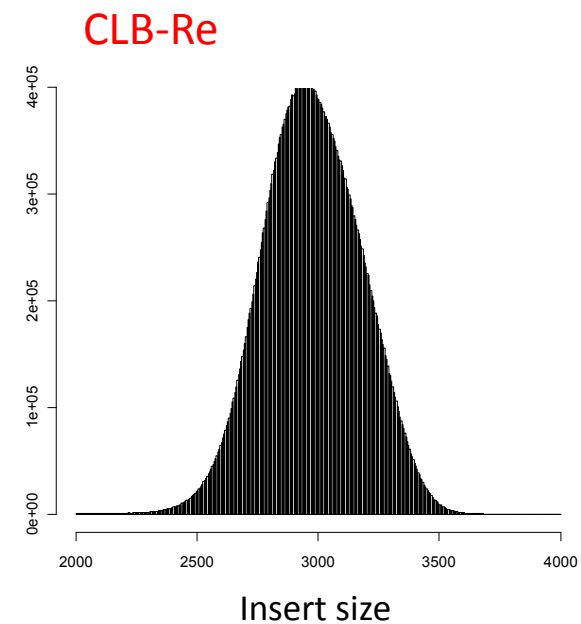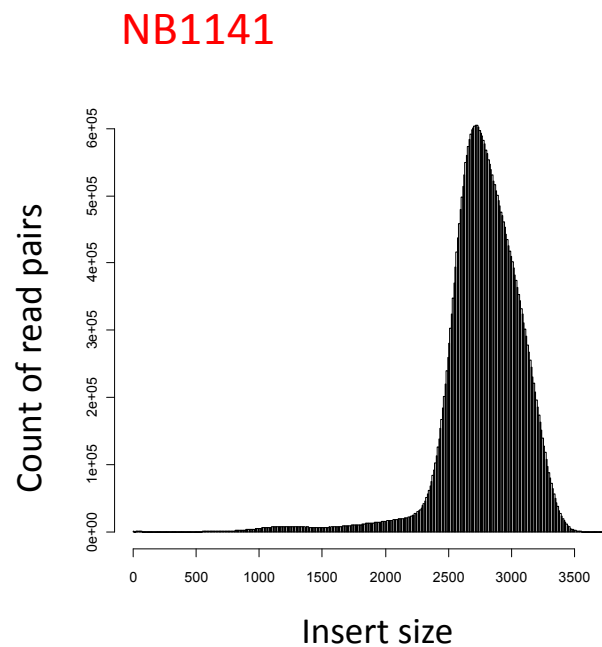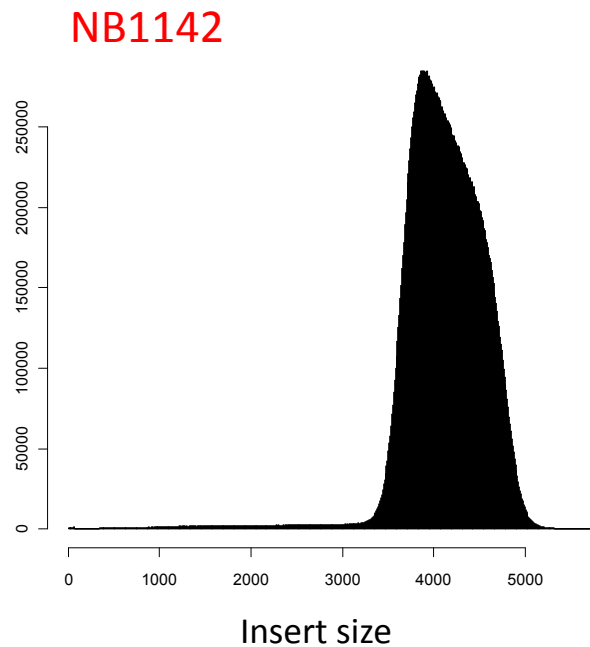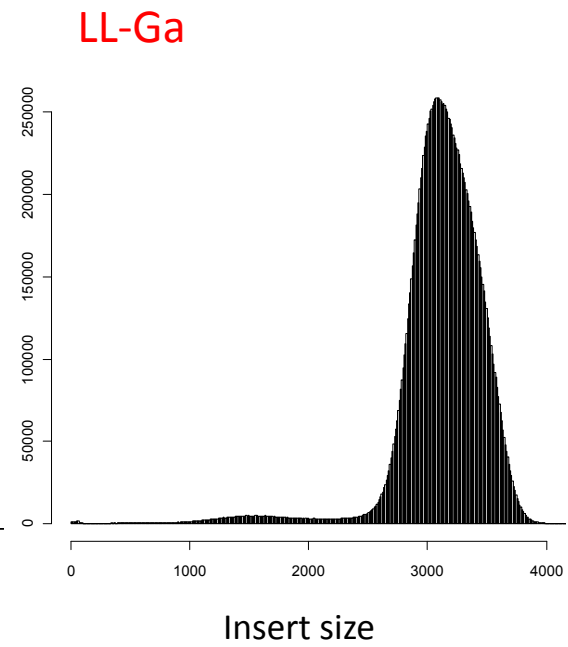

Supplement: Figure S10 — Observed distribution of the insert sizes. (PDF) [file pone.0072182.s010.pdf]
