# Supplementary material for: Breakpoint Features of Genomic Rearrangements in Neuroblastoma with Unbalanced Translocations and Chromothripsis
Source: PLoS One. 2013 Aug 26;8(8):e72182. doi: 10.1371/journal.pone.0072182 (PMC3753337; doi:10.1371/journal.pone.0072182)
Supplement: Figure S8 — Analysis workflow for detection of copy number alterations and structural variants using whole genome sequencing data. (PDF) [file pone.0072182.s008.pdf]

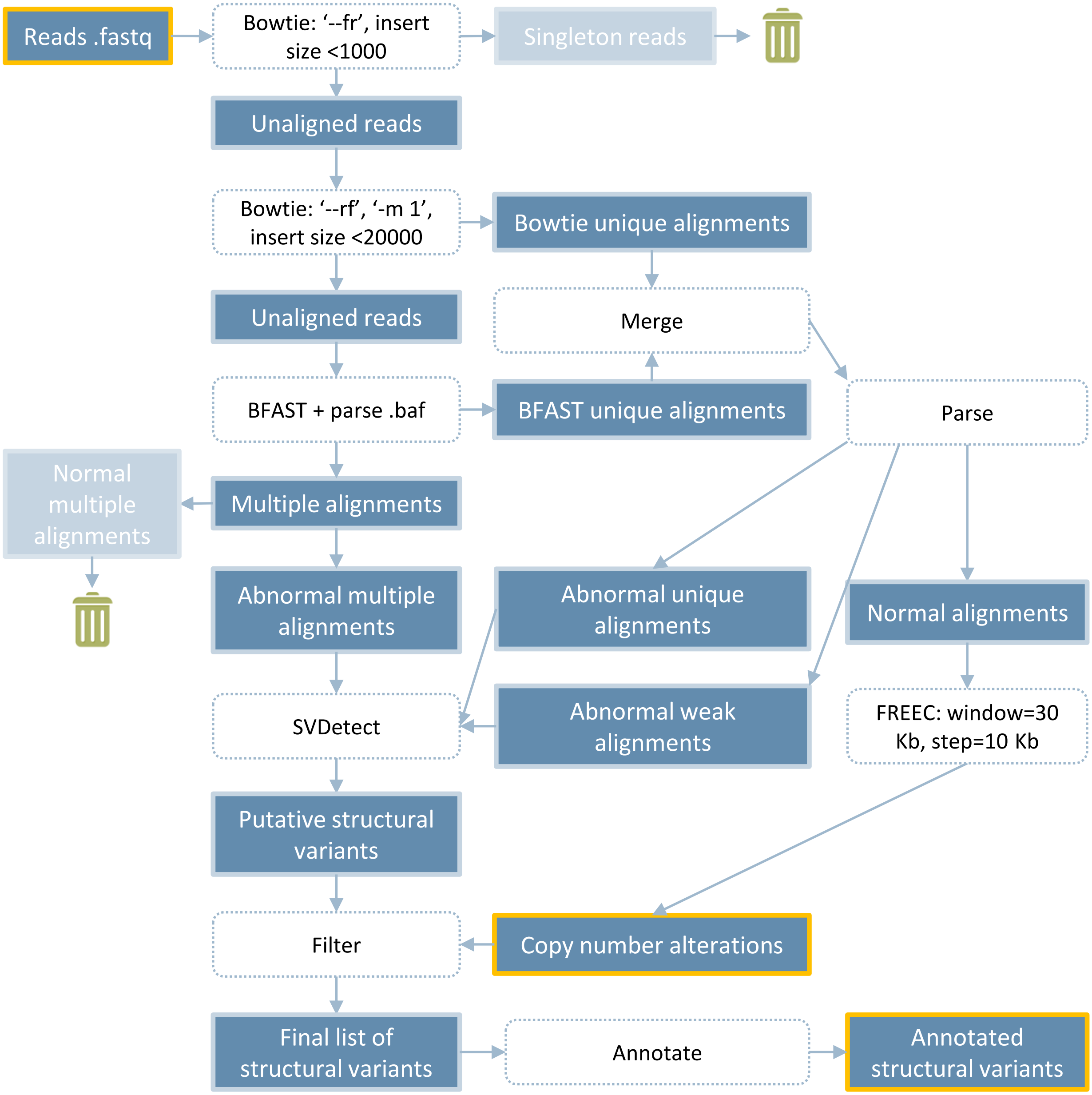

Supplementary Figure S8. Analysis workflow for detection of copy number alterations and structural variants using whole genome sequencing data.
