# Supplementary material for: Breakpoint Features of Genomic Rearrangements in Neuroblastoma with Unbalanced Translocations and Chromothripsis
Source: PLoS One. 2013 Aug 26;8(8):e72182. doi: 10.1371/journal.pone.0072182 (PMC3753337; doi:10.1371/journal.pone.0072182)
Supplement: Figure S9 — Annotation of predicted structural variants (SVs) with genomic information. Gene exons are shown by large rectangles (dark blue and purple), introns are shown by thin arrows, 5′ and 3′ UTRs are shown by small rectangles (dark blue and purple). A read pair representative of the SV signature is shown by two connected rectangles (light blue) with white arrows showing the direction of sequencing. We say that a link falls in a gene if any of corresponding reads falls between the gene transcription start site (TSS) and transcription end. If reads fall within a region up to 2 kb upstream gene TSS, we annotate the corresponding end of the link as falling in a promoter region. (PDF) [file pone.0072182.s009.pdf]

Supplementary figure S9: Annotation of predicted structural variants (SVs) with genomic information. Gene exons are shown by large rectangles (dark blue and purple), introns are shown by thin arrows, 5’ and 3’ UTRs are shown by small rectangles (dark blue and purple). A read pair representative of the SV signature is shown by two connected rectangles (light blue) with white arrows showing the direction of sequencing. We say that a link falls in a gene if any of corresponding reads falls between the gene transcription start site (TSS) and transcription end. If reads fall within a region up to 2 kb upstream gene TSS, we annotate the corresponding end of the link as falling in a promoter region.

SV annotation — Gene A — Gene B — Gene annotations

Intergenic

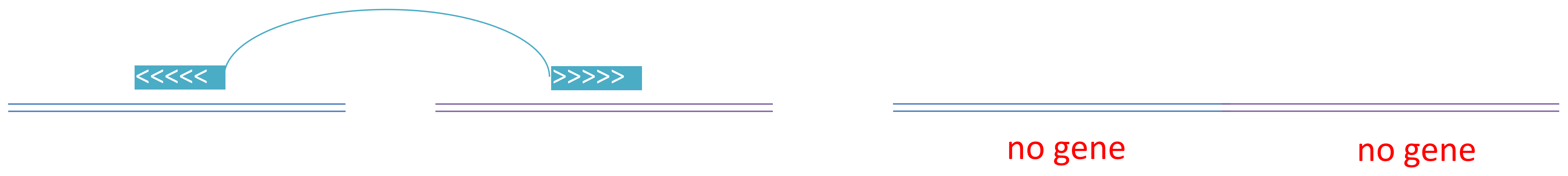

SV does not contain mate pairs mapping between transcription start site (TSS) and transcription end (TE) of any gene

Truncated

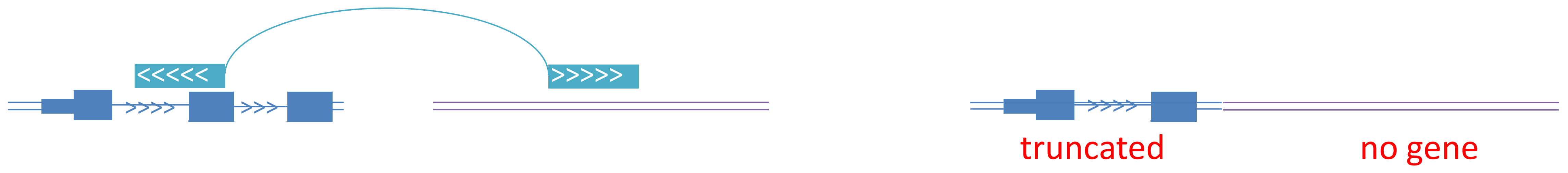

Reads map between TSS and TE of gene A in orientation that suggests that the 5’ part of the gene is kept, while 3’ part is lost. An intergenic region is joined to the the 5’ part of the gene probably resulting in a stop codon, and thus in a truncated form of protein A.

Truncated

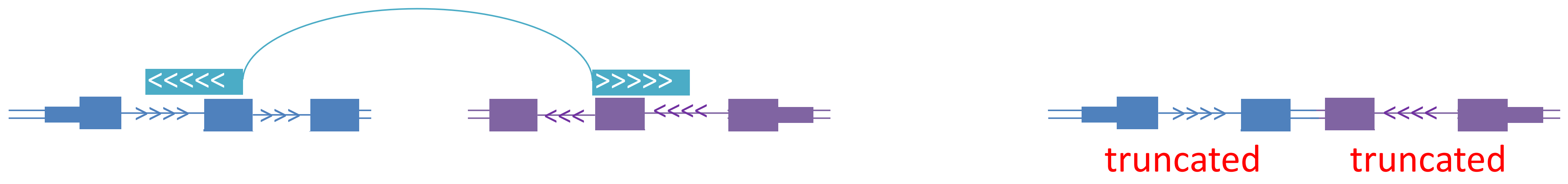

Reads map between TSS and TE of genes A and B in orientation that suggests that the 5’ parts of genes A and B are kept, while 3’ parts are lost. This SV can results in two possibly overlapping transcripts. Reverse strand transcription probably results in stop codons, and thus in a truncated form of proteins A and B.

Possible chimera

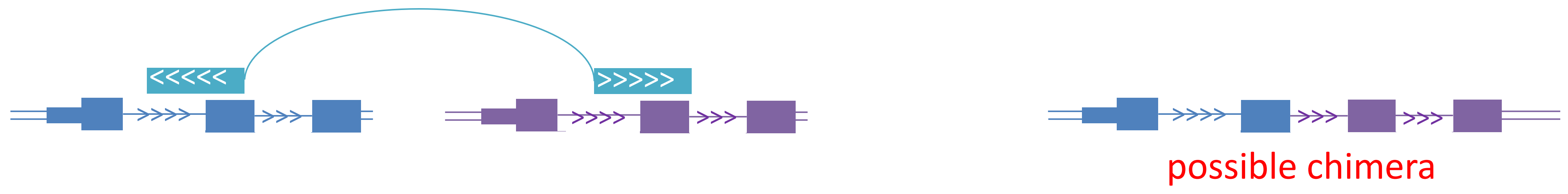

Reads map between TSS and TE of genes A and B in orientation that suggests that the 5’ part of gene A is kept as well as 3’ part of gene B, while 3’ part of gene A is lost as well as 5’ part of gene B. This SV can results in a chimeric transcript starting at TSS of gene A and ending at TE of gene B.

No change of function

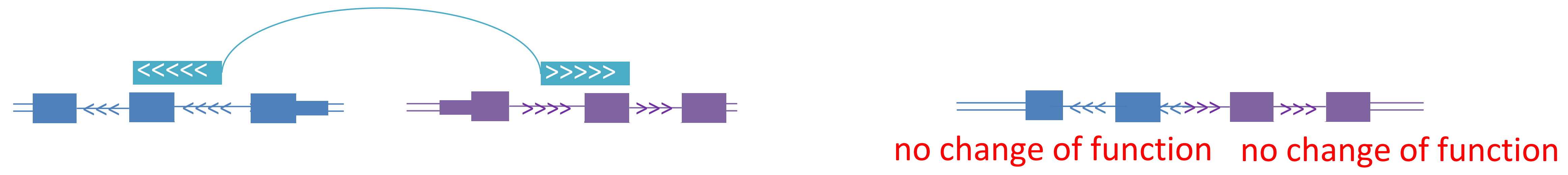

Reads map between TSS and TE of genes A and B in orientation that suggests that the 3’ parts of genes A and B are kept and 5’ parts of genes A and B are lost. Since both promoters of genes A and B are lost, this SV does not result in any abnormal transcript.

No change of function

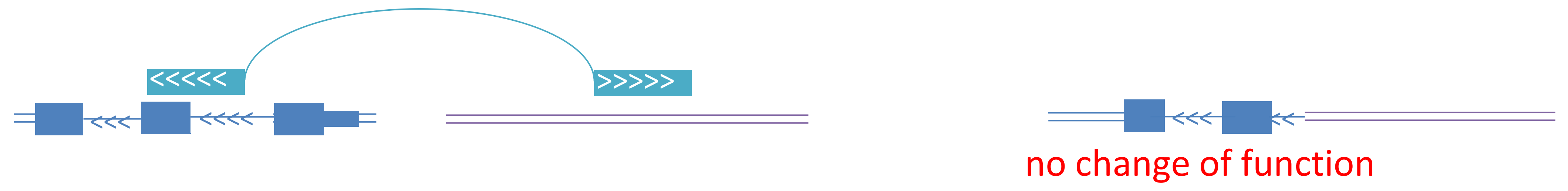

Reads map between TSS and TE of gene A in orientation that suggests that the 3’ part of gene A is kept and the 5’ part is lost. Since gene A loses its promoter, this SV does not result in any abnormal transcript.

May change function

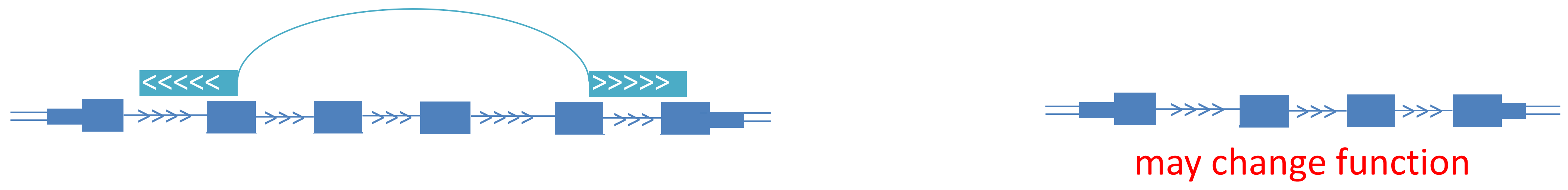

Reads map between TSS and TE of gene A in orientation that suggests that there is a deletion, duplication or inversion of a part of the gene. If any of the gene isoforms has one or more exons in the deleted/inverted/duplicated area, we annotate this SV as an SV that “may change gene function”. However, we keep in mind that this(-ese) exon(-s) may be spliced out in normal cells, and thus this SV does not always change gene function even is the rearranged form is expressed.

May not change function

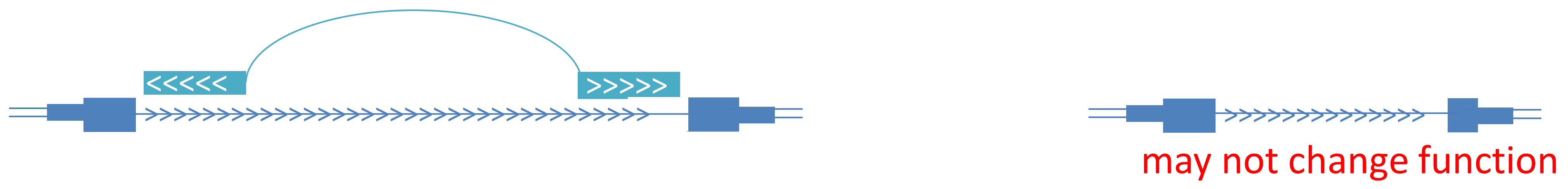

Reads map between TSS and TE of gene A in orientation that suggests that there is a deletion, duplication or inversion of a part of the gene. There is no isoform with an exon in the deleted/inverted/duplicated area, thus we annotate this SV as an SV that “may not change gene function”. However, we keep in mind that even an SV in an intron may have functional consequences, for example when it creates a new splice site or modify binding sites of regulatory proteins.
