# Supplementary material for: Breakpoint Features of Genomic Rearrangements in Neuroblastoma with Unbalanced Translocations and Chromothripsis
Source: PLoS One. 2013 Aug 26;8(8):e72182. doi: 10.1371/journal.pone.0072182 (PMC3753337; doi:10.1371/journal.pone.0072182)
Supplement: Table S1 — Read statistics (total number of sequencing reads, aligned reads, insert size distibution, normal and abnormal reads). (PDF) [file pone.0072182.s011.pdf]

Supplementary table S1. Read statistics (total number of sequencing reads, aligned reads, insert size distibution, normal and abnormal reads).

**Whole genome mate-pair sequencing**	**LL-Ga**	**CLB-Ga**	**CLB-Re**	**NB1141**	**NB1142**
# total pairs	71 603 080	70 905 689	69 021 650	79 727 107	69 415 514
"FR", <1kb, Bowtie	29 026 577 (40.54%)	2 995 641 (4.22%)	3 630 991 (5.26%)	3 685 331 (4.62%)	8 098 970 (11.67%)
"RF", <20kb, Bowtie	17 697 433 (24.72%)	43 147 211 (60.85%)	42 203 093 (61.14%)	37 406 575 (46.92%)	28 689 042 (41.33%)
Selected range for "normal" reads (start-to-start, nt)	2056 -3858	2178-3538	2206-3591	2022-3461	2144-5148
Median (nt)	3 154	2 953	2 972	2 794	4 120
All "normal" pairs (Bowtie+BFAST)	26 617 582	52 848 333	51 106 325	56 878 957	45 653 192
All "abnormal" pairs (Bowtie+BFAST)	6 793 628	8 600 870	8 220 980	5 455 524	4 445 782
All "normal" pairs w/o duplicates	13 315 914	20 180 194	15 972 090	30 463 767	21 580 472
All "abnormal" pairs w/o duplicates	2 154 642	1 347 141	1 174 494	1 314 957	905 879

**RNA sequencing**	**LL-Ga**	**CLB-Ga**	**CLB-Re**	**NB1141**	**NB1142**
# total pairs	N/A	427 309 163	424 746 931	421 827 293	347 009 368
# pairs with both ends mapped	NA	176 036 292	176 009 335	162 088 194	121 304 777
# pairs with mapping Q > 4, w/o duplicates	NA	11 460 153	12 051 382	11 927 431	3 986 691
