# Supplementary material for: Breakpoint Features of Genomic Rearrangements in Neuroblastoma with Unbalanced Translocations and Chromothripsis
Source: PLoS One. 2013 Aug 26;8(8):e72182. doi: 10.1371/journal.pone.0072182 (PMC3753337; doi:10.1371/journal.pone.0072182)
Supplement: Table S3 — Intra-chromosomal rearrangements predicted by SVDetect with insert size <50 kb. (PDF) [file pone.0072182.s013.pdf]

Supplementary Table S3: Intra-chromosomal rearrangements predicted by SVDetect with insert size < 50kb.

	CLB-Ga (% in DGV*)	CLB-Re (% in DGV)	NB1141 (% in DGV)	NB1142 (% in DGV)
Intra-chromosomal links with an insert size < 50 kb	364 (40%)	435 (50%)	164 (49%)	160 (57%)
DELETION	231 (30%)	242 (51%)	59 (69%)	47 (68%)
INS_FRAGMT	3 (67%)	3 (33%)	5 (60%)	3 (33%)
INSERTION	101 (58%)	137 (58%)	56 (18%)	61 (57%)
INV_DUPLI	1 (0%)	0	0	0
INV_INS_FRAGMT	1 (0%)	0	1 (0%)	0
INV_FRAGMENT	0	2 (0%)	0	1 (0%)
INVERSION	0	5 (0%)	1 (0%)	0
LARGE_DUPLI	2 (50%)	4 (0%)	3 (66%)	4 (50%)
TRANSLOC	25 (56%)	42 (36%)	39 (64%)	44 (48%)
Intergenic	194	247	99	109
Promoter	2	5	1	2
Short form	9	11	3	5
May change function	23	11	1	0
Possible chimera	1	4	0	0
May not change function	126	147	57	42
Does not change function	9	10	3	2

DELETION	deletion of a fragment
INSERTION	insertion of a unknown fragment
INV_INS_FRAGMT	insertion of a short fragment (fragment location is known, fragment is inverted)
INS_FRAGMT	insertion of a short fragment (fragment location is known)
INV_DUPLI	inverted duplication
INV_FRAGMENT	inversion (both ends of the inversion are confirmed by read pairs)
INVERSION	inversion (only one end of the inversion is confirmed by read pairs)
LARGE_DUPLI	large duplication (size of the duplicated fragment is greater than average insert size)
TRANSLOC	translocation

* both breakpoint regions of the SV fall close to the ends of an annotated Deletion/Invertion/Duplication in the Database of Genomic Variants (DGV)
