# Supplementary material for: Breakpoint Features of Genomic Rearrangements in Neuroblastoma with Unbalanced Translocations and Chromothripsis
Source: PLoS One. 2013 Aug 26;8(8):e72182. doi: 10.1371/journal.pone.0072182 (PMC3753337; doi:10.1371/journal.pone.0072182)
Supplement: Table S5 — Theoretical and observed frequencies of microhomology on validated breakpoint junctions in chromothripsis and non-chromothripsis cases. (PDF) [file pone.0072182.s015.pdf]

Supplementary Table S5: Theoretical and observed frequencies of microhomology on validated breakpoint junctions in chromothripsis and non-chromothripsis cases.

Count / # common base pairs	Random frequency	Non-chromothripsis	Chromothripsis
**0**	75.0%	4 (24%)	12 (32%)
**1**	18.8%	3 (18%)	9 (24%)
**2**	4.7%	5 (29%)	6 (16%)
**3**	1.2%	2 (12%)	3 (8%)
**≥4**	0.4%	3 (18%)	8 (21%)
**Chi-square p-value**		1.41E-36	4.34E-96
