# Supplementary material for: Breakpoint Features of Genomic Rearrangements in Neuroblastoma with Unbalanced Translocations and Chromothripsis
Source: PLoS One. 2013 Aug 26;8(8):e72182. doi: 10.1371/journal.pone.0072182 (PMC3753337; doi:10.1371/journal.pone.0072182)
Supplement: Methods S1. — (PDF) [file pone.0072182.s017.pdf]

SUPPLEMENTARY METHODS

Genome-wide massively parallel sequencing to characterize genomic rearrangements in neuroblastoma: from unbalanced translocations to chromothripsis

Valentina Boeva, Stéphanie Jouannet, Romain Daveau, Valérie Combaret, Cécile Pierre-Eugène, Alex Cazes, Caroline Louis-Brennetot, Gudrun Schleiermacher, Sandrine Ferrand, Gaëlle Pierron, Alban Lermine, Thomas Rio Frio, Virginie Raynal, Gilles Vassal, Emmanuel Barillot, Olivier Delattre and Isabelle Janoueix-Lerosey

MATE-PAIR SEQUENCING

The DNA libraries were prepared following Illumina protocol "Preparing 2–5 kb Samples for Mate Pair Library Sequencing" using 10 micrograms of genomic DNA (Supplementary Methods). In brief, 10 micrograms of genomic DNA was fragmented by a controlled nebulization targeted to shear the high molecular weight DNA into smaller fragments. The fragmented DNA was end-repaired with biotin labeled dNTPs. This step was followed by a gel size selection (around 3 kb). The size selected fragments were circularized and sheared again by nebulization to a fragment length of around 400 bp. Fragments with biotin labels were affinity purified. Purified fragments were end-repaired, A-tailed, and ligated to Illumina Paired-end sequencing adapters. The adapter-ligated libraries were amplified with 18 PCR cycles. Gel size selection was carried out to select for fragments in the range of 400–600 bp. The libraries were ready for paired-end cluster generation on a flow cell (4 pM/lane DNA was applied to the flow cell). The Illumina Genome Analyzer IIx system generated paired-end sequences of 35, 50 or 76 nucleotides. Images from the instrument were processed using the manufacturer's software to generate FASTQ sequence files. The sequencing resulted in about 70 million raw paired reads per sample (Supplementary table 1).

RNA SEQUENCING

100 ng of polyA mRNAs (22 ng for NB1142) were then fragmented by incubation with RNAse III according to the SOLiD® Total RNA-Seq Kit instructions (Life Technologies, protocol Part Number 4452437 Rev. B, July 2011). Fragmented RNAs were further purified using the RiboMinus™ Concentration Module (Invitrogen, Carlsbad, Ca). The yield and size distribution of the fragmented RNA were assessed using the Quant-iT™ RNA assay kit (Invitrogen, Carlsbad, Ca) and the RNA 6000 Pico Chip kit (Agilent, Santa Clara, Ca). Fragmented RNAs were hybridized and ligated with the SOLiD™ adaptator mix and reverse transcribed according to supplier instructions. The isolated cDNAs were size-selected around 200 bp using 2 rounds of Agencourt® AMPure® XP Reagent (Beckman Coulter) and amplified according to the SOLiD® Total RNA-Seq Kit protocol (15 PCR cycles for CLB-Ga, CLB-Re and NB1141; 18 PCR cycles for NB1142). The yield and size distribution of the cDNAs were assessed using the Quant-iT™ HS DNA assay kit and the High Sensitivity DNA Assay Chip Kit on the Agilent® 2100 Bioanalyzer. Templated beads were generated for sequencing by SOLiD™ EZ Bead™ System automates

using standard manufacturers' protocols. The four samples were multiplexed and a total of 500 pM with a titration of 0.4 pM were used for emulsion, amplification and enrichment steps.

DATA ALIGNMENT

WHOLE GENOME MATE-PAIR DATA. The NB1141 and NB1142 libraries were run on a single lane of Illumina flow cell resulting in 76 nt mate-pair reads. The CLB-Ga and CLB-Re were each run on two lanes resulting in 35 and 50 nt mate-pair reads.

Since we used mate-pair protocol, sequenced read pairs were expected to be oriented outwards (reverse-forward orientation, "RF"). Insert size was expected to be about 3,000 bp (see Suppl. Fig. S10 for observed distributions of insert size lengths). We also expected to have some contamination by non-biotinylated fragments (forward-reverse orientation, "FR", and <1000 nt insert size). Thus, we first aligned the reads with Bowtie v0.12.7[1] using paired-end mode ('--fr') with insert size <1000 nt to filter out read pairs corresponding to non-biotinylated fragments. During this step, from 4 to 41% of raw reads were discarded from further analysis (Supplementary Table 1). Reads which were not mapped to the reference genome using the above options were then realigned to hg19 using Bowtie's mate-pair mode ('--rf', insert size <20,000 nt, unique match '-m 1', up to three mismatches in the seed '-n 3'). The alignment rate varied from 42 to 65%. Using Bowtie alignment we could evaluate normal insert size distribution and divide aligned reads into "normal" and "abnormal unique" categories according to insert-size (the central 99.8% of insert size distribution corresponds to "normal" reads). We calculated the median insert size of sequenced DNA fragments using paired-ends reads mapping to the genome in the correct orientation (Supplementary table 1). The obtained values (3154 bp for LL-Ga, 2953 bp for CLB-Ga, 2972 bp for CLB-Re, 2794 bp for NB1141, and 4120 bp for NB1142) suggested that the size selection of fragments was accurate.

We then performed more sensitive, gapped alignment with BFAST v6.3.[2] We parsed the BFAST output (.baf) to keep the best alignment per read and also to tag reads as "normal", "abnormal unique", "abnormal multiple" and "abnormal weak". We merged the output of the two tools. This resulted in 27 to 57 million "normal" reads and in 4 to 9 million "abnormal" reads (Supplementary Table 1). We filtered out PCR duplicates (±2nt at least for one start out of two) using in-house software.

RNA-SEQ DATA. Alignment of RNA-seq data was performed by the SOLiD Lifescope v.2.5 software to hg19 and a library of exon junctions provided by Lifescope. We filtered out low mapping quality reads (threshold 5). We also filtered out PCR duplicates (the same start and end position for the two ends) using in-house software. This resulted in 4 to 12 million reads (Supplementary Table 1).

ANNOTATION OF COPY NUMBER STATUS

To annotate copy-number, we used FREEC v3.9.[3] Since we had from 13 to 30 million reads per sample after duplicate filtering, we decided to use a sliding window of 30 kb with a step of 10 kb. This resulted in more than 100 reads per window in case of the normal genotype. We discarded windows with less than 60% of uniquely mappable positions (using theoretical mappability profiles constructed for 50-nt reads with 2 mismatches with the GEM toolbox,

http://sourceforge.net/apps/mediawiki/gemlibrary). We called a copy number alteration (CNA) only if at least two consecutive windows supported the CNA.

We showed that for the tumor samples, from 3.3 to 31% of the genome were found to have an abnormal copy number status (Suppl. Fig. S3).

PREDICTION OF STRUCTURAL VARIANTS

We ran the SVDetect[4] to detect links predictive for structural variants (translocations, insertions, deletions, tandem duplications, etc.) using "abnormal" reads for each sample. We applied the following parameters: window size = 7500 nt, step length = 500 nt, nb_pairs_threshold = 1, nb_pairs_order_threshold = 1, strand filtering = TRUE, order_filtering = TRUE, insert size filtering = TRUE, indel_sigma_threshold = 1, dup_sigma_threshold = 1, final score threshold = 0.1, singleton sigma threshold=4. We used the values of median and standard deviation calculated for each sample using "normal" reads.

We then processed the output of SVDetect:

- We merged overlapping links that corresponded to structural variants (SVs) of the same type in the same sample.
- We kept a link only if it was specific* to one sample. The reason to do so is that (1) there is no common copy number breakpoints for any two samples (according to copy-number analysis by FREEC) and (2) such filtering allows eliminating many artifact links – for example, this filter discarded 94% of inter-chromosomal links with more than 3 pairs per link from LL-Ga (blood) sample.
- In some samples, up to 30% of intra-chomosomal links were annotated as "Small duplications". This is an artifact due to issues in DNA library preparation. Thus, we discarded all links annotated as "Small Duplications" from further analysis.
- We applied filters to keep links that were confirmed by at least four read pairs and that contain at least 85% of pairs whose orientation and order were consistent with the SV type.
- We discarded all links that contained only multiple hits or weak alignments and, at the same time, did not correspond to any change of copy number status predicted by FREEC (up to 50 kb).
- We discarded links that fell close to poly-N non-assembled telomeric/centromeric regions of hg19 (up to 1 Mb).
- The length of fragments in DNA libraries did not exceed 4000 bp for all samples but NB1142. Thus, we discarded links for which read clusters were larger than 4000 bp (5500 bp for NB1142).
- We also discarded links with too short read clusters (<500 bp).
- We discarded links read clusters of which contained satellite sequences (≥300 bp of length) of the same type at both ends of the link.
- We eliminated links both ends of which fell on "random", "hap", "Un" or "M" chromosomes.
- We eliminated links for which read clusters contained regions detected as putative genomic duplications within the golden path[5], when this link could be explained by misalignment of reads.

The post-processing of the SVDetect output resulted in very modest number of predicted SV (Table 2).

* We consider a link to be specific to sample A, when it does no overlap links in other samples or when the link in A corresponds to a putative amplicon structure (>80 pairs) and in other samples only few pairs are mapped in the corresponding regions (up to 3 pairs).

ANNOTATION OF PREDICTED SVS

We considered a link falling in a gene if any of corresponding reads fell between the gene transcription start site (TSS) and transcription end. A SV in a gene can have different consequences such as loss or gain of function, chimeric gene, etc. Thus, we annotated each gene with the following tags: "Truncated" when the SV can result in a truncated form of the protein, "Possible chimera" when the SV can result in a formation of a chimeric gene, "Does not change function" when the 3' end of the gene is gained or amplified and is not involved in a chimeric gene or the 5' end is deleted and this SV does not lead to a chimeric gene, "May change function" when the SV falls within a gene and duplicates, inverses or deletes exons, and "May not change function" when the SV falls in an intronic region of the gene (Supplementary figure 9). If reads fell within a region up to 2 kb upstream gene TSS, we annotated the corresponding end of the link as falling in a promoter region. In the annotation, we do not take into account the size of gene chunks resulting from the SV. For example, if a 5' end of the gene is deleted as a result of an SV, we annotate the gene as "truncated" regardless the size of the deleted part.

BREAKPOINT ANALYSIS

Motifs implicated in formation or repair of double strand DNA breaks or corresponding to particular DNA structures (such as putative triple helix)[6, 7] were searched at immediate or short proximity of each breakpoint using the Fuzznuc software tool (http://mobyle.pasteur.fr/cgi-bin/portal.py?#forms::fuzznuc) (Supplementary table 6). We considered the ± 20 bp and ± 125 bp windows surrounding each breakpoint as immediate or short proximity, respectively. In order to evaluate the significance of a potential enrichment of these motifs, we counted the same motifs in paired control sequences defined as ±500 bp and ±5 kb extended regions around each breakpoint without overlapping it, at immediate or short proximity, respectively. Breakpoint and control sequences were also compared with respect to GC, polypurine, polypyrimidine and alternating purine/pyrimidine contents. All comparisons were evaluated using a Chi2-based two-tailed test of equal proportions. Yate's continuity correction was applied whenever possible and stringent Bonferroni correction was used to adjust p-values. A usual threshold of 5% was considered as statistically significant.

REFERENCES

1. Langmead, B., et al., *Ultrafast and memory-efficient alignment of short DNA sequences to the human genome.* Genome Biol, 2009. **10**(3): p. R25.
2. Homer, N., B. Merriman, and S.F. Nelson, *BFAST: an alignment tool for large scale genome resequencing.* PLoS One, 2009. **4**(11): p. e7767.
3. Boeva, V., et al., *Control-free calling of copy number alterations in deep-sequencing data using GC-content normalization.* Bioinformatics, 2011. **27**(2): p. 268-9.

4. Zeitouni, B., et al., *SVDetect: a tool to identify genomic structural variations from paired-end and mate-pair sequencing data.* Bioinformatics, 2010. **26**(15): p. 1895-6.
5. Bailey, J.A., et al., *Recent segmental duplications in the human genome.* Science, 2002. **297**(5583): p. 1003-7.
6. Abeysinghe, S.S., et al., *Translocation and gross deletion breakpoints in human inherited disease and cancer I: Nucleotide composition and recombination-associated motifs*. Hum. Mutat., 2003. **22**(3): p. 229-244.
7. Storlazzi, C.T., et al., *Gene amplification as double minutes or homogeneously staining regions in solid tumors: origin and structure*. Genome Res., 2010. **20**(9):p. 1198-1206.
